# Monocyte Preprogramming by Tobacco Carcinogens and Fructose Intake Accelerates Lung Cancer Progression via Metabolic and Epigenetic Pathways

**DOI:** 10.7150/ijbs.125622

**Published:** 2026-03-25

**Authors:** Jee Hwan Ahn, Hye-Young Min, Jisung Kim, Xuan Wei, Namwon Kim, Ho-Young Lee

**Affiliations:** 1 Department of Molecular Medicine and Biopharmaceutical Sciences, Graduate School of Convergence Science and Technology and College of Pharmacy, Seoul National University, Seoul 08826, Republic of Korea.; 2 Natural Products Research Institute, Seoul National University, Seoul 08826, Republic of Korea.; 3 College of Pharmacy, Seoul National University, Seoul 08826, Republic of Korea.; 4 Research Institute of Pharmaceutical Sciences, Seoul National University, Seoul 08826, Republic of Korea.

**Keywords:** tobacco carcinogens, non-small cell lung cancer, cancer progression, metabolic reprogramming, fructose

## Abstract

Although tobacco smoking is the leading cause of lung cancer (LC), excessive sugar intake has also emerged as a potential risk factor, yet its mechanistic contribution remains poorly defined. In this study, we investigated how high-fructose intake modulates the tobacco carcinogen-induced LC progression. Co-exposure to 4-(methylnitrosamino)-1-(3-pyridyl)-1-butanol and benzo[a]pyrene (NNK and BaP; collectively referred to as NB) combined with a high-fructose diet markedly accelerated tumor progression in multiple mouse models, including Kras^G12D/+^-driven LC and LKB1-deficient (LKB1^KO^) lung tumors. NB-induced LC progression was suppressed by restricting glucose metabolism, indicating a metabolic dependency. Mechanistically, NB exposure stimulated transcriptional programs that promote monocyte/macrophage recruitment within the tumor microenvironment and enhanced fructose uptake through both transcriptional and post-transcriptional upregulation of fructose transporters, including glucose transporter 8 (GLUT8). This metabolic reprogramming increased acetylation of histones and signal transducer and activator of transcription 3 (STAT3), leading to transcriptional upregulation of genes governing macrophage differentiation and M2 polarization. Analysis of human LC samples revealed enrichment of pro-metastatic IL-10^+^ and VEGFA^+^ M2 macrophages, which correlated with poor clinical outcomes. Collectively, these findings demonstrate that NB-driven fructose metabolism induces epigenetic reprogramming of macrophages to promote LC progression and identify pro-metastatic M2 macrophages as potential prognostic biomarkers and therapeutic targets.

## Introduction

Lung cancer (LC) is the leading cause of cancer-related death globally, with tobacco smoking (TS) as the primary risk factor 1. TS induces metabolic syndrome and disrupts carbohydrate metabolism, thereby promoting the growth and aggressiveness of various tumor types, including LC 2, 3. Excessive carbohydrate intake is associated with cancer progression 4, while glycolytic inhibition by 2-deoxy-D-glucose (2DG) impairs tumor growth and metastasis 2, 5. Potent tobacco carcinogens, such as 4-(methylnitrosamino)-1-(3-pyridyl)-1-butanol (NNK) and benzo[a]pyrene (BaP), activate tumor-promoting pathways via nicotinic acetylcholine receptors (nAChRs), β-adrenergic receptors (β-ARs), or the aryl hydrocarbon receptor (AhR) 6. We previously showed that exposure to NNK and BaP (NB) induces hyperglycemia and accelerates LC progression, whereas 2DG treatment suppresses these effects in mice 2. Thus, targeting carbohydrate metabolism offers a promising strategy to counter TS-induced LC progression.

Tumors of various histological origins display increased macrophage infiltration during the early stages of cancer progression 7. While some tissue-resident macrophages derive from adult monocytes, most originate from fetal progenitors 8. Circulating monocytes differentiate into macrophages under the control of transcription factors (TFs) such as MAFB, MAF, and early growth response protein 1 (EGR1) 9, adopting either anti-tumoral M1 or pro-tumoral M2 phenotypes based on environmental cues 10. M2 macrophages comprise subtypes (M2a-M2d) that promote tissue repair, immunosuppression, fibrosis, and metastasis through cytokines like transforming growth factor-beta (TGF-β), interleukin-10 (IL-10), and vascular endothelial growth factor (VEGF) 11. Glucose metabolism regulates macrophage differentiation and polarization 12. M1 macrophages depend on glycolysis 13, whereas M2 macrophages rely on oxidative phosphorylation and exhibit higher glucose uptake than naïve macrophages 14-16. TF activation and environmental signals shape macrophage subtype specification 8. Tumor-associated macrophages (TAMs) predominantly show M2-like, pro-tumor functions 16. We previously showed that NB exposure increases glucose uptake in TAMs by upregulating glucose transporters (GLUT1 and GLUT3), thereby promoting LC progression in mice 2. However, the role of fructose and the mechanisms by which carbohydrates regulate macrophage polarization in NB-induced LC remain unclear.

In this study, we investigated how fructose supplementation influences NB-induced LC progression, with a particular focus on macrophage differentiation and polarization. We demonstrate that NB exposure enhances fructose uptake in TAMs by upregulating fructose transporters, including GLUT8, thereby facilitating LC progression. Furthermore, NB promotes monocyte differentiation and polarization toward a pro-tumoral M2 phenotype under conditions of excessive fructose availability. Mechanistically, excess fructose availability drives an epigenetic program in which increased acetylation of histones and STAT3 activates transcriptional networks governing macrophage differentiation and M2 polarization. Collectively, our findings suggest that metabolic targeting of dysregulated macrophages represents a promising strategy to mitigate TS-associated LC progression.

## Materials and Methods

### Reagents

Antibodies against acetylated signal transducer and activator of transcription 3 (STAT3) (Ac-STAT3 at K685, cat. no. 2523), STAT3 (cat. no. 9139), arginase 1 (ARG1, cat. no. 93668), liver kinase B1 (LKB1, cat. no. 3047), and TGF-β (cat. no. 3711) were purchased from Cell Signaling Technology (Danvers, MA, USA). Anti-β-actin (cat. no. sc-47778) and anti-TTF1 (cat. no. sc-13040) antibodies were purchased from Santa Cruz Biotechnology (Dallas, TX, USA). The anti-F4/80 antibody (cat. no. MCA497G) was acquired from Bio-Rad Laboratories (Hercules, CA, USA). The anti-CD68 antibody (cat. no. MA5-13324) was purchased from Thermo Fisher Scientific (Waltham, MA, USA). Antibodies against myeloperoxidase (MPO, cat. no. ab9535), inducible nitric oxide synthase (iNOS, cat. no. ab178945), IL-10 (cat. no. ab34843), and VEGFA (cat. no. ab46154) were purchased from Abcam (Cambridge, UK). Antibodies against acetyl-histone H3-K9 (H3K9ac, cat. no. A7255), acetyl-histone H3-K27 (H3K27ac, cat. no. A7253), trimethyl-histone H3-K9 (H3K9me3, cat. no. A2360), and trimethyl-histone H3-K27 (H3K27me3, cat. no. A2363) were acquired from ABclonal Technology (Woburn, MA, USA). The anti-GLUT8 antibody (cat. no. bs-4241R) was purchased from Bioss (Woburn, MA, USA). The anti-CCL22/MDC antibody (cat. no. MAB336) was acquired from R&D Systems (Minneapolis, MN, USA). Horseradish peroxidase (HRP)-conjugated secondary antibodies (anti-mouse: cat. no. CNG004-0005; anti-rabbit: cat. no. CNG005-0005) were purchased from CellNest (Hanam-si, Gyeonggi-do, Republic of Korea). Stemregenin 1 (SR1, S, cat. no. S2858) and Stattic (Sta, cat. no. S7024) were acquired from Selleck Chemicals (Houston, TX, USA). Mecamylamine hydrochloride (mecamylamine, M, cat. no. 2843) was purchased from Tocris Bioscience (Bristol, UK). NNK (cat. no. M325750) was purchased from Toronto Research Chemicals (Toronto, Ontario, Canada). 2DG (cat. no. D6134), 3-bromopyruvate (3BP, bromopyruvic acid, cat. no. 16490), BaP (cat. no. B1760), (±)-propranolol hydrochloride (propranolol, P, cat. no. P0884), urethane (cat. no. U2500), tamoxifen (TM, cat. no. T5648), corn oil (cat. no. C8267), collagenase (type IV, cat. no. C5138), DNase I (type IV, cat. no. D5025), hyaluronidase (type V, cat. no. H6254), 4',6-diamidino-2-phenylindole dihydrochloride (DAPI, cat. no. D9542), dimethyl sulfoxide (DMSO, cat. no. D4540), and other chemicals unless otherwise indicated were purchased from Sigma-Aldrich (St. Louis, MO, USA).

### Cell culture

A549 and Lewis lung carcinoma (LLC) cells were purchased from the American Type Culture Collection (ATCC, Manassas, VA, USA). L929 and LA-4 cells were obtained from the Korean Cell Line Bank (Seoul, Republic of Korea), and THP-1 cells were provided by Dr. Kyu-Won Kim (Seoul National University, Seoul, Republic of Korea). Cells were cultured in RPMI 1640 medium (cat. no. LM011-01, Welgene, Gyeongsan-si, Republic of Korea; for A549, L929, LA-4, and THP-1 cells) or Dulbecco's modified Eagle's medium (DMEM, cat. no. LM001-05, Welgene; for LLC cells) at 37°C in a humidified environment containing 5% CO_2_. The media were supplemented with 10% fetal bovine serum (FBS, cat. no. S001-07, Welgene) and 1× antibiotic-antimycotic solution (antibiotics, cat. no. LS203-01, Welgene). All human cell lines were authenticated and validated using the AmplFLSTR Identifier PCR Amplification Kit (Applied Biosystems, Foster City, CA, USA; cat. no. 4322288) in 2014. Cells were used within two months after recovery from cryopreserved, validated stocks or from cryopreserved purchased cells, and all cultures were confirmed to be mycoplasma-free.

For *in vitro* experiments examining the mechanism of NB-induced regulation of differentiation and M2 macrophage-associated factors, THP-1 cells were exposed to NB (a combined treatment of 1 μM NNK and 1 μM benzo[a]pyrene [BaP]) under standard culture conditions (RPMI 1640 medium containing 2 g/L glucose and supplemented with 10% FBS and antibiotics) for three days. In experiments involving fructose supplementation, cells were treated with NB under low-glucose conditions (RPMI 1640 medium [cat. no. R1383, Sigma-Aldrich] supplemented with 2 g/L sodium bicarbonate [cat. no. S5761, Sigma-Aldrich], 0.8 g/L D-glucose [cat. no. G7021, Sigma-Aldrich], 10% FBS, and antibiotics) with the addition of 10 g/L D-fructose (cat. no. F0127, Sigma-Aldrich). For inhibitor studies, THP-1 cells were treated with NB under standard culture conditions for three days, and the indicated inhibitors were added during the final 24 h of NB exposure.

### Animal studies

All procedures for the maintenance of mouse models and mouse experiments were performed in accordance with protocols approved by the Institutional Animal Care and Use Committee (IACUC) of Seoul National University. *Stk11*^tm1.1Sjm^/J (*Stk11*^fl/fl^, Lkb1 KO) and 129S/Sv-*Kras*^tm3Tyj^/J (*Kras*^G12D/+^, Kras LA2) mice were purchased from Jackson Laboratory (Bar Harbor, ME, USA). *Sftpc*-CreER^T2^ mice were kindly provided by Dr. Brigid L. M. Hogan (Department of Cell Biology, Duke University School of Medicine, Durham, NC, USA). *Kras*^G12D/+^, *Stk11*^fl/fl^, and *Sftpc*-CreER^T2^ mice with C57BL/6 backgrounds were backcrossed with FVB mice over seven generations. *Stk11*^fl/fl^ mice were crossed with *Sftpc*-CreER^T2^ mice to generate the *Sftpc*-CreER^T2^/*Stk11*^fl/fl^ mice. The mice were maintained in a specific-pathogen-free facility. Mouse experiments were performed as previously described 2. All mice were included in the experiment using the *Kras*^G12D/+^ mouse model. In the subcutaneous allograft model, mice that had been inoculated with the cells but not exhibited subcutaneous tumor development were excluded.

To investigate the dietary effects on spontaneous lung tumor growth and metastasis, 2-month-old *Kras*^G12D/+^ mice were fed a low-carbohydrate diet (LCD; cat. no. TD.06414, Envigo, Indianapolis, IN, USA) or an LCD supplemented with high fructose corn syrup (LCD/HFCS) and were exposed to vehicle (Veh) or NB (a combined treatment of 3 μmol NNK and 3 μmol BaP dissolved in corn oil and administered by oral gavage) for one month. HFCS was prepared by combining D-glucose (cat. no. G8270, Sigma-Aldrich) and D-fructose (cat. no. F0127, Sigma-Aldrich) in a 45:55 ratio using distilled water, and the mice were allowed free access to 25% HFCS in their drinking water. Tumor growth was monitored using a Quantum GX2 microCT Imaging System and Quantum GX2 4.0 Software (PerkinElmer, Alameda, CA, USA). The multiplicity, volume, and burden of tumors were microscopically evaluated, as described in our previous report 17. The number and size of the tumors in five sections uniformly distributed throughout each lung were calculated to determine tumor multiplicity, volume, and burden.

For the subcutaneous allograft model, an *Stk11* knockout (KO) murine primary lung tumor cell subclone derived from tamoxifen-treated and urethane-treated *Sftpc*-CreER^T2^;*Stk11*^fl/fl^ mice (LKB1^KO^ cells) was used. The detailed method used for primary culture is described in the following section. LKB1^KO^ cells were suspended in 100 μL of PBS containing 25% Matrigel (cat. no. 354234, Corning, Corning, NY, USA) per spot and 6 × 10^5^ cells were subcutaneously inoculated with the right flank of FVB mice. After 1.5 weeks, mice that harbored palpable tumors (tumor volume ≥ 50 mm^3^) were randomly assigned into several groups and fed an LCD or LCD/HFCS. The mice were also treated with NB by oral gavage twice a week for four weeks. When necessary, 3BP (10 mg/kg, dissolved in PBS) was orally administered once a day and five days a week for one month. Tumor growth was monitored by measuring the short and long diameters of the tumors using calipers in a blinded manner. Tumor volume was calculated using the following formula: (short diameter)^2^ × (long diameter) × 0.5. According to institutional ethical guidelines, mice bearing subcutaneous tumors exceeding 15-17 mm in diameter or 10% of body weight (approximately 2,000 mm^3^ in volume) were euthanized. We confirmed that the maximal tumor size in our experiments did not exceed these ethical limits.

### Primary culture of murine tumor cells

1-month-old *Sftpc*-CreER^T2^;*Stk11*^fl/fl^ mice were orally administered tamoxifen (3 mg/mouse dissolved in corn oil at a concentration of 25 mg/mL) for five consecutive days to induce Cre recombinase. After three months, the mice were treated with urethane (1 g/kg) via intraperitoneal injection once a week for four weeks to facilitate tumorigenesis. 2.5 months after the final urethane treatment, the mice were euthanized via deep anesthesia using isoflurane inhalation, and the lung tumors were excised. Tumors were dissociated by incubating with a digestion buffer (1 mg/mL type IV collagenase, 0.1 mg/mL type V hyaluronidase, and 20 units/mL type IV DNase I in Hank's balanced salt solution [cat. no. H6648, Sigma-Aldrich]) for 20 min at 37°C. After passing the tumor in a digestion buffer through a 70 μm cell strainer (cat. no. 93070, SPL Life Sciences, Gyeonggi-do, Republic of Korea), cells were pelleted by centrifugation at 800 × *g* for 7 min. Red blood cells (RBCs) were removed from the cell pellets by incubation with 1× RBC lysis buffer (cat. no. 420301, BioLegend, San Diego, CA, USA). After centrifugation, the cells were cultured in RPMI 1640 supplemented with 10% FBS and antibiotics. A subclone with loss of LKB1 expression was used for phenotype evaluation, such as cell proliferation, anchorage-dependent colony formation, and migration/motility. Single-cell suspensions from tumors (*Kras*^G12D/+^ mouse lung tumors or subcutaneous L6 allograft-derived tumors) were obtained using the same procedures. The isolated cells were cultured in RPMI 1640 medium supplemented with 10% FBS and antibiotics, and the viable cells were utilized for further experiments.

### Isolation of bone marrow-derived macrophages

Cells were isolated from the bone marrow of 8-10-week-old C57BL/6 mice from the femurs and tibias. After lysing the red blood cells with 1× RBC lysis buffer, the remaining bone marrow cells were cultured in RPMI 1640 containing 10% heat-inactivated FBS, antibiotics, and 10% L929 conditioned medium for differentiation. During maturation, cells were treated with NB.

### Generation of *in vitro* conditioned media derived from macrophages

To generate macrophage-conditioned media (CM), macrophages treated with Veh or NB, either alone or together with 10 g/L fructose (Fru), were cultured in serum-free media for 24-36 h. The supernatant was harvested, filtered with a 0.45 μm filter, and stored at -20°C. To generate CM-exposed cancer cells, lung cancer cells were incubated with CM (25% CM in the culture medium) for the indicated time periods. 25% CM was changed three times per week.

### Immunohistochemistry analysis

Immunohistochemistry analysis was performed using an anti-TTF1 antibody. Sections of formalin-fixed and paraffin-embedded (FFPE) tissue were deparaffinized, rehydrated, and subjected to antigen retrieval using a citrate-based antigen unmasking solution (cat. no. H-3300, Vector Laboratories, Burlingame, CA, USA). After permeabilization with 0.3% Triton X-100 in Tris-buffered saline containing 0.1% Tween 20 (TBST), the slides were incubated with a blocking buffer (5% normal serum in TBST) for 1 h at room temperature (RT). Slides were incubated first with anti-TTF1 primary antibodies diluted in TBST containing 3% bovine serum albumin (BSA, cat. no. A0100-005, GenDEPOT, Baker, TX, USA) (1:100) overnight at 4°C, then washed three times with TBST. After treatment with 0.3% hydrogen peroxide solution, the slides were incubated with a biotinylated secondary antibody (Vector Laboratories) diluted in TBST containing 3% BSA (1:500) for 1 h at RT. Solutions A and B (VECTASTAIN Elite ABC-HRP kit, cat. no. PK-6100, Vector Laboratories) were added simultaneously for 30 min, and signals were detected using a 3,3'-diaminobenzidine (DAB) substrate kit (HIGHDEF DAB chromogen/substrate set, cat. no. ENZ-ACC105, Enzo Life Sciences, Farmingdale, NY, USA). The slides were counterstained with hematoxylin.

### Immunofluorescence staining

For frozen tissue and cell staining, sections of optimal cutting temperature (OCT)-embedded tissue and cells on glass coverslips were fixed in 4% paraformaldehyde for 15 min at RT. After permeabilization with 0.2% Tween 20 or 0.3% Triton X-100 in TBST, immunofluorescence staining was performed as previously described 18 using antibodies against MPO (Abcam, 1:200 dilution), F4/80 (Bio-Rad, 1:100 dilution), ARG1 (Cell Signaling, 1:100 dilution), and iNOS (Abcam, 1:200 dilution). Slides were counterstained with DAPI.

To determine changes in the expression or subcellular localization of GLUT8 following treatment with NNK and BaP, either alone or in combination, THP-1 cells were treated with vehicle (DMSO, final concentration ≤ 0.1%), NNK (1 μM), BaP (1 μM), or their combination (NB) for three days. THP-1 cells were collected, washed with PBS, and cytocentrifuged onto silane-coated glass slides at 800 rpm for 5 min using 5 × 10⁴ cells per spot. Cells were air-dried briefly, fixed with 4% paraformaldehyde in PBS for 15 min at RT, washed with PBS, and permeabilized with 0.1% Triton X-100 in PBS for 10 min. Non-specific binding was blocked with 3% BSA in PBS for 1 h at RT, followed by incubation with anti-GLUT8 antibody (1:200 in blocking buffer) overnight at 4 °C in a humidified chamber. After PBS washes, cells were incubated with fluorophore-conjugated secondary antibodies diluted in blocking buffer for 1 h at RT in the dark and counterstained with DAPI.

### Anchorage-independent colony formation assay

Lung cancer cells (5 × 10^3^ cells/well) were mixed with low-melting agar solution (final 0.4%; top agar) and 500 μL of cell suspension was poured onto 500 μL of 1% base agar that had solidified in 24-well plates prior to the experiment. After solidification of the top agar, more than 500 μL of culture medium in the absence or presence of the treatment was added to the agar and incubated for two weeks. Colonies were stained with a 500 μg/mL MTT solution and photographed. Colonies were counted using the ImageJ software (ver. 1.53k, National Institutes of Health, Bethesda, MD, USA).

### Anchorage-dependent colony formation assay

The cells were plated in 6-well plates at a density of 300 cells per well or 12-well plates at a density of 100 cells per well. Following a two-week incubation, colonies were fixed using 100% methanol, stained with a 0.02% crystal violet solution, and either photographed or counted manually.

### Scratch assay

Cells were cultured on 6-well plates. Confluent cells were scraped in a linear fashion using a sterile tip to generate a scratch, followed by washing with medium to eliminate cell debris. Cells were imaged immediately following the development of a scratch and at various intervals up to 36 h using the EVOS FL Cell Imaging System (Thermo Fisher Scientific).

### Fructose uptake assay

Cells were incubated with NBD-fructose (20 μM, cat. no. 9002314, Cayman Chemical, Ann Arbor, MI, USA) for 30 min at 37 °C. After the NBD-fructose-containing medium was removed, the cells were washed and suspended in PBS. The fluorescence intensity was measured using a SpectraMax M3 microplate reader (Molecular Devices, San Jose, CA, USA).

### Real-time polymerase chain reaction (PCR)

Total RNA was isolated using the easy-BLUE total RNA extraction kit (cat. no. 17061, Intron Biotechnology, Seongnam, Gyeonggi-do, Republic of Korea) or RNAIso Plus (cat. no. 9108, Takara Bio Inc., Kusatsu, Shiga, Japan) according to the manufacturer's instructions. Complementary DNA (cDNA) was synthesized using the First-Strand cDNA Synthesis kit (cat. no. AT301, TransGen Biotech, Beijing, China). All real-time PCR assays were performed on a QuantStudio^TM^ 5 Real-Time PCR System (Thermo Fisher Scientific) using a SYBR Green-based qPCR master mix (cat. no. RT500M, Enzynomics, Daejeon, Republic of Korea) and gene-specific primers. The following thermocycler conditions for real-time PCR were applied: pre-incubation at 95 °C for 15 min; 40-45 cycles of 95 °C for 10 sec, 60 °C for 20 sec, and 72 °C for 30 sec; and a final melt curve analysis to determine reaction specificity. Relative quantification of mRNA expression was performed using the comparative CT (cycle threshold) method described in a previous publication 19. The primer sequences are listed in **Table S1**.

### Western blot analysis

Cells were harvested with modified RIPA lysis buffer (50 mM Tris-HCl [pH 7.5], 150 mM NaCl, 1 mM EDTA, 0.25% sodium deoxycholate, 1% Triton X-100, 1 mM Na_3_VO_4_, 100 mM NaF, 0.5 mM dithiothreitol [DTT], 1 mM phenylmethylsulfonyl fluoride [PMSF], 1 μg/mL aprotinin, 1 μg/mL leupeptin, and 20 mM β-glycerophosphate). The lysates were centrifuged at 13,000 rpm for 30 min at 4 °C and the supernatant was collected (whole cell lysates). The protein concentrations of prepared whole-cell lysates were measured using BCA assay reagents. Equal amounts of protein were then separated by 8-10% SDS-PAGE and transferred onto PVDF membranes. The membranes were blocked with blocking buffer (3% BSA in TBST containing 0.02% sodium azide) for 1 h at RT. They were then incubated with a primary antibody (1:1,000 dilution in blocking buffer) overnight at 4°C. Afterward, the membranes were washed four times with TBST for 1 h at RT and incubated with the corresponding secondary antibody (1:5,000 dilution in 3% skim milk in TBST). The membranes were washed four times with TBST for 1 h at RT. Blots were visualized using an enhanced chemiluminescence (ECL) detection kit (cat. no. BWF0100, Biomax, Guri-si, Gyeonggi-do, Republic of Korea).

### Chromatin immunoprecipitation (ChIP) assay

The ChIP assay was performed using the SimpleChIP^®^ Enzymatic Chromatin IP Kit (cat. no. 9002S, Cell Signaling Technology) according to the manufacturer's instructions. For immunoprecipitation, antibodies against H3K9ac (Abcam, 1:500 dilution), STAT3 (Cell Signaling Technology, 1:200 dilution), or negative control (normal IgG antibody, Cell Signaling Technology, 1:200 or 1:500 dilution) were incubated with cross-linked chromatin overnight at 4°C with agitation. Precipitated DNA enrichment was analyzed by real-time PCR using primer sequences (**Table S2**) and normalized to the respective 2% input.

### Transwell migration assay

For the Transwell (8.0 μm pore size, Corning) migration assay, the outer membrane was coated with 0.05% gelatin. Cells that were exposed to NB, either alone or in combination with D-fructose (10 g/L), were seeded in the lower compartments (2 × 10^5^ cells/well). After a day, THP-1 cells that were exposed to NB either alone or in combination with D-fructose were seeded onto the upper wells (1 × 10^5^ cells/well) in RPMI 1640 medium without serum, and then the plates were further incubated for 18 h at 37°C in an incubator humidified with 5% CO_2_. After incubation, the membrane was stained with a hematoxylin solution and mounted onto a glass slide. The number of stained cells per field was counted using a Nuance microscope (PerkinElmer).

### Human tissue samples

Human lung tissue microarray (TMA) was purchased from TissueArray.Com (cat. no. LC821, Derwood, MD, USA). The TMA comprised 70 cores from 35 lung cancer patients and 10 cores from healthy donors with a history of smoking (2015 WHO classification) 20. Tissues were analyzed by immunofluorescence staining using antibodies against IL-10 (Abcam, 1:100 dilution), VEGFA (Abcam, 1:100 dilution), and CD68 (Thermo Fisher Scientific. 1:100 dilution) to determine IL-10 or VEGFA expression in CD68^+^ tumor-infiltrating macrophages. Fluorescence images were captured using a Zeiss LSM700 confocal microscope (Carl Zeiss AG, Oberkochen, Germany) and quantified using the ZEN Imaging Software (Carl Zeiss AG).

### *In silico* analysis

We used a publicly available dataset (GSE31210) 21 deposited in the Gene Expression Omnibus (GEO, National Center for Biotechnology Information Bethesda, MD, USA). Gene expression levels and clinical information for each patient sample were manually downloaded and analyzed using GraphPad Prism (ver. 10.4, GraphPad Software, San Diego, CA, USA). Gene set enrichment analysis (GSEA) was conducted using the GSEA software (Broad Institute, Massachusetts Institute of Technology, Cambridge, MA, USA) according to the previous report 22. GSEA utilized a custom gene set group comprising 15 gene sets associated with oncogenic signatures and cancer progression, sourced from the Molecular Signatures Database 23. The GLUT8^high^ and GLUT8^low^ or STAT3^high^ and STAT3^low^ groups for GSEA were defined based on the median value of the data. The survival rates of patients with lung cancer were analyzed using Prognoscan online software (https://dna00.bio.kyutech.ac.jp/PrognoScan/) 24, and Kaplan-Meier curves were drawn using GraphPad Prism after retrieving the data table from the Prognoscan software and dividing the high and low expression groups using Prognoscan analysis results. Genes that overlapped with at least two prognostic gene signatures for both lung adenocarcinoma and squamous cell carcinoma were defined as prognosis-associated genes 25. The correlation between GLUT8 or STAT3 expression and the expression of each prognostic signature gene was determined using Spearman's rank correlation analysis. The following probes were used to obtain gene expression levels: SLC2A8: 218985_at; STAT3: 208992_s_at; TPX2: 210052_s_at; KIAA0101: 202503_s_at; RRM2: 209773_s_at; MCM2: 202107_s_at; PRC1: 218009_s_at; FGFR2: 208229_at; CDKN3: 209714_s_at; UBE2C: 202954_at; GPC3: 243243_at.

### Statistics

The data are presented as the mean ± SD. All experiments were independently performed at least twice, and representative results are shown. Values presented in the graphs were generated using multiple replicates of a representative experiment. Statistical significance was determined by a two-tailed Student's *t*-test, Mann-Whitney test, one-way analysis of variance (ANOVA), or the Kruskal-Wallis test using GraphPad Prism (ver. 10.4). The Shapiro-Wilk test was performed to determine whether the *in vitro* or *in vivo* data followed a normal distribution. *p*-values less than 0.05 were considered significant.

## Results

### Dietary fructose promotes NB-induced growth and metastasis of LC cells, including those harboring KRAS or LKB1 mutation

**Figure 1:**
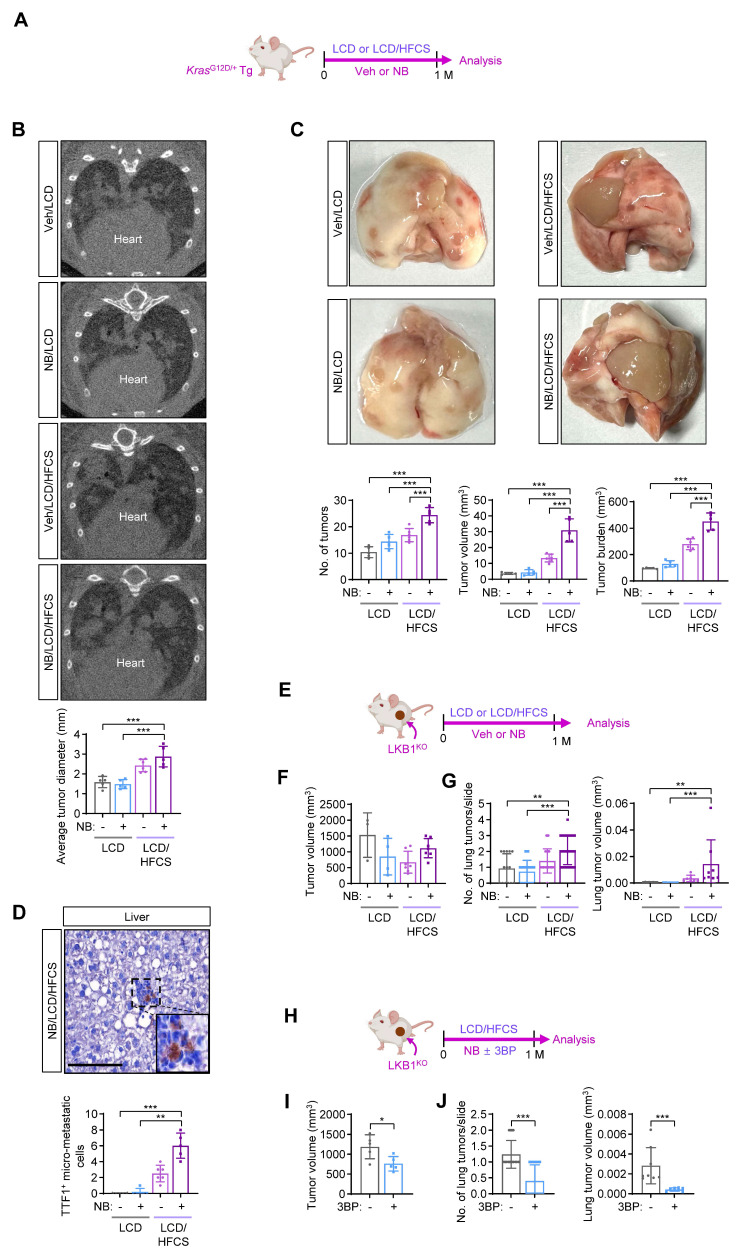
** Dietary sugar promotes NB-induced lung cancer progression.** (**A**) Schematic diagram of the experimental protocol using the *Kras*^G12D/+^ transgenic (Tg) mouse model. (**B**) Representative micro-CT images of the lungs and quantification of the lung tumors identified using micro-CT images (mean ± SD,* n* = 5-6). (**C**) Representative images of whole lung tissues and multiplicity, volume, and burden of the lung tumors (mean ± SD,* n* = 5-6). (**D**) Representative images of H&E-stained liver tissues and quantification of the micrometastasis of lung tumors in the liver (mean ± SD,* n* = 5-6). Scale bar, 50 μm. (**E**,** H**) Schematic diagram of the experimental protocol using subcutaneous LKB1^KO^ allografts. (**F**,** I**) The tumor volume of primary tumors of LKB1^KO^ cells in the indicated groups (mean ± SD,* n* = 3-7). (**G**,** J**) Tumor multiplicity (mean ± SD,* n* = 15 or 25) and volume (mean ± SD,* n* = 8) in the lungs. *p < 0.05, **p < 0.01, and ***p < 0.001, as determined by one-way ANOVA with Dunnett's multiple-comparison test (**B, C**, **F**), the Kruskal-Wallis test with Dunn's multiple-comparison test (**D**, **G**), a two-tailed Student's t-test (**I**), and Mann-Whitney test (**J**).

Non-small cell lung cancer (NSCLC) patients frequently harbor mutations in the *KRAS* oncogene and the tumor suppressor LKB1 (encoded by the *STK11* gene), both of which regulate glucose metabolism 26. These mutations are associated with increased tumor viability, aggressiveness, and chemoresistance 27, 28. To determine whether high-fructose intake promotes NB-induced LC progression in the context, we used *Kras*^G12D/+^ transgenic (Tg) mice (Kras^G12D^) 29 exposed to Veh or NB under the conditions of LCD or LCD/HFCS: Kras^G12D^-Veh/LCD, Kras^G12D^-NB/LCD, Kras^G12D^-Veh/LCD/HFCS, and Kras^G12D^-NB/LCD/HFCS (**Fig. 1A**). Micro-CT analysis showed a significantly greater number of lung tumor nodules in the Kras^G12D^-NB/LCD/HFCS group compared to the others (**Fig. 1B**). Although all groups showed 100% tumor incidence, histological analysis revealed markedly increased tumor multiplicity and burden in the Kras^G12D^-NB/LCD/HFCS group (**Fig. 1C**; **Fig. S1A**). Immunohistochemistry analysis of liver tissues using a TTF1 antibody, a marker for lung adenocarcinoma 30, demonstrated significantly more TTF1-positive clusters in the Kras^G12D^-NB/LCD/HFCS group, suggesting enhanced metastatic spread, which is rare in aged Kras^G12D^ mice 29(**Fig. 1D**).

To evaluate the effect of high-fructose intake on NB-induced lung cancer progression in the context of LKB1 loss, we generated LKB1-deficient lung cancer cells using a urethane-induced tumor model 31. *Sftpc*-CreER^T2^;*Stk11*^fl/fl^ mice, in which LKB1 was knocked out in type 2 alveolar epithelial cells following tamoxifen treatment, were administered urethane to induce tumorigenesis (**Fig. S1B**). Visible lung tumors developed by 3.5 months post-injection. Primary lung tumor cells were cultured, and a subclone with complete LKB1 loss (LKB1^KO^) was selected, showing no LKB1 expression compared to LA-4 cells—derived from urethane-induced tumors in wild-type mice 32 (**Fig. S1C**). LKB1^KO^ cells exhibited significantly enhanced proliferation, colony formation, and migration (**Figs. S1D-F**). We next established FVB mice bearing subcutaneous LKB1^KO^ tumors and exposed them to Veh or NB for one month either LCD or LCD/HFCS: LKB1^KO^-Veh/LCD, LKB1^KO^-NB/LCD, LKB1^KO^-Veh/LCD/HFCS, or LKB1^KO^-NB/LCD/HFCS (**Fig. 1E**). Primary tumor growth did not differ significantly among groups (**Fig. 1F**). However, H&E staining of lung tissues revealed the highest tumor multiplicity and volume in the LKB1^KO^-NB/LCD/HFCS group (**Fig. 1G; Fig. S1G**). In a follow-up experiment, LKB1^KO^ tumor-bearing mice were exposed to NB and LCD/HFCS, with or without 3BP—a glycolytic inhibitor targeting hexokinase and glyceraldehyde-3-phosphate dehydrogenase (GAPDH) 33(**Fig. 1H**). 3BP treatment significantly reduced NB-induced tumor growth (**Fig. 1I**) and lung metastasis (**Fig. 1J**). These results indicate that high-fructose intake promotes NB-induced lung cancer progression, likely through enhanced glycolysis.

### High-fructose intake and NB exposure synergistically promote macrophage recruitment to the TME and enhance pro-tumoral activity

**Figure 2:**
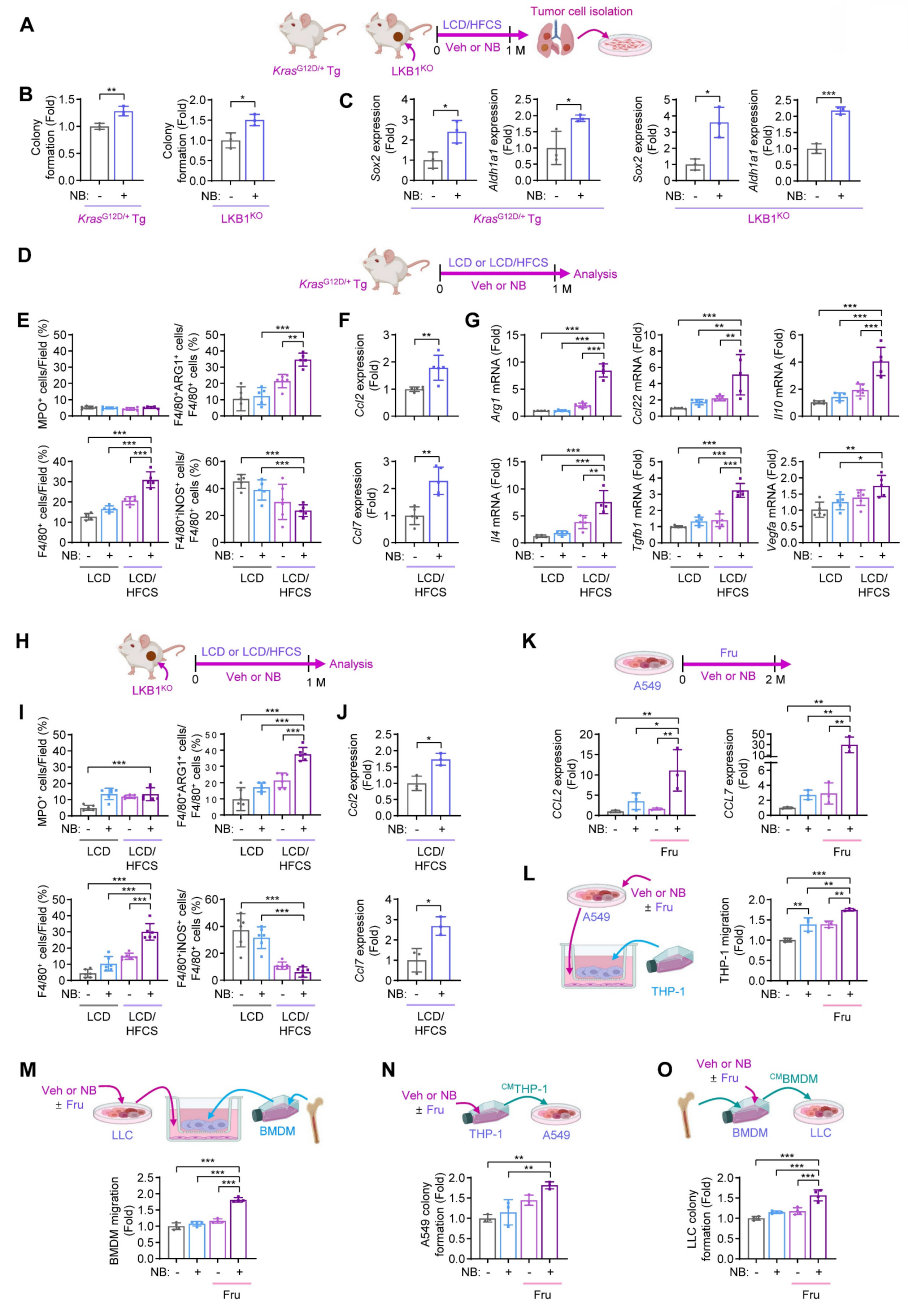
** Fructose supplementation enhances NB-induced monocytes/macrophage recruitment and tumor-promoting functions.** (**A-C**) Primary cultures of *Kras*^G12D/+^ transgenic (Tg) mouse lung tumor cells and LKB1^KO^ allograft tumor cells were derived from Veh/LCD/HFCS and NB/LCD/HFCS mice. (**A**) Schematic diagram of the experimental design. (**B**) Anchorage-dependent colony formation (mean ± SD,* n* = 3). (**C**) mRNA expression of *Sox2* and *Aldh1a1* (mean ± SD,* n* = 3). (**D-J**) Immunofluorescence (IF) and real-time PCR analyses using the *Kras*^G12D/+^ Tg mouse model and subcutaneous LKB1^KO^ allografts. (**D, H**) Schematic diagram of the experimental protocol. (**E, I**) Quantitative analysis of the immunofluorescence images assessing infiltration of the indicated stromal cells in *Kras*^G12D/+^ Tg mouse lung tumors (**E**, mean ± SD,* n* = 5 or 6) and LKB1^KO^ allograft tumors (**I,** mean ± SD,* n* = 6). (**F, J**) Real-time PCR analysis of *Ccl2* and *Ccl7* expression in *Kras*^G12D/+^ Tg mouse lung tumors (**F**, mean ± SD,* n* = 5) and LKB1^KO^ allograft tumors (**J**, mean ± SD,* n* = 3). (**G**) Real-time PCR analysis of the indicated genes in *Kras*^G12D/+^ Tg mouse lung tumors (mean ± SD,* n* = 5 or 6). (**K**) Real-time PCR analysis of *CCL2* and *CCL7* in A549 cells treated with vehicle (Veh, DMSO) or NB (1 μM NNK plus 1 μM BaP), with or without D-fructose (10 g/L) for two months (mean ± SD, *n* = 3). (**L, M**) Migration assays of THP-1 cells (**L**, mean ± SD, *n* = 3) or bone marrow-derived macrophages (BMDMs) (**M**, mean ± SD, *n* = 4) incubated with cancer cells treated with Veh or NB with or without fructose. (**N, O**) Anchorage-independent colony formation of A549 cells (**N**, mean ± SD, *n* = 3) or LLC cells (**O**, mean ± SD, *n* = 4) exposed to conditioned media (CM) from Veh- or NB-treated macrophages in the presence or absence of fructose. *p < 0.05, **p < 0.01, and ***p < 0.001, as determined by a two-tailed Student's t-test (**B, C**,** F**,** J**) and one-way ANOVA with Dunnett's multiple-comparison test (**E**,** G, I**,** K-O**).

To investigate how NB exposure promotes LC progression in a fructose-rich environment, we analyzed tumor cells from Kras^G12D^-Veh/LCD/HFCS, Kras^G12D^-NB/LCD/HFCS, LKB1^KO^-Veh/LCD/HFCS, and LKB1^KO^-NB/LCD/HFCS groups (**Fig. 2A**). Primary cultures of LC cells from Kras^G12D^-NB/LCD/HFCS and LKB1^KO^-NB/LCD/HFCS groups exhibited increased colony formation and elevated mRNA expression of cancer stem cell (CSC)-related genes, including *Sox2* and *Aldh1a1*, compared to their respective vehicle-treated controls (**Figs. 2B, C**).

We previously showed that NB exposure combined with high-glucose intake enhances infiltration of M2-type macrophages, promoting LC progression 2. To determine whether a similar effect occurs under high-fructose conditions, we performed immunofluorescence (IF) analysis on tumor tissues from Kras^G12D^-NB/LCD/HFCS and the corresponding control groups (**Fig. 2D**). We first assessed levels of MPO^+^ neutrophils, CD4^+^ T cells, CD8^+^ T cells, and F4/80^+^ macrophages, including arginase 1 (ARG1)^+^ M2-type macrophages (F4/80^+^ARG1^+^) and inducible nitric oxide synthase (iNOS)^+^ M1-type macrophages (F4/80^+^iNOS^+^). The Kras^G12D^-NB/LCD/HFCS group exhibited increased infiltration of total and M2-type macrophages (F4/80^+^ARG1^+^) but not M1 macrophages (F4/80^+^iNOS^+^), compared to Kras^G12D^-Veh/LCD, Kras^G12D^-NB/LCD, and Kras^G12D^-Veh/LCD/HFCS groups (**Fig. 2E**; **Fig. S2A**). However, the recruitment of MPO^+^ neutrophils, CD4^+^ T cells, and CD8^+^ T cells was minimally modulated in the Kras^G12D^-NB/LCD/HFCS group (**Fig. 2E**; **Figs. S2A, B**). Real-time PCR analyses further revealed that the Kras^G12D^-NB/LCD/HFCS group exhibited elevated expression of *Ccl2* and *Ccl7*, key chemokines involved in monocyte recruitment 34 (**Fig. 2F**), and M2-associated anti-inflammatory mediators, including *Arg1*, *Ccl22*, *Il10*, *Il4*, *Tgfb1*, and *Vegfa*, but not M1-associated pro-inflammatory mediators (*Il1b*, *Il6*, *Tnf*, *Nos2*, and *Ifng*), compared to the other groups (**Fig. 2G; Fig. S2C**). We also performed IF analysis on tumor tissues from LKB1^KO^-NB/LCD/HFCS and the corresponding control groups (**Fig. 2H**). Similarly, LKB1^KO^-NB/LCD/HFCS group exhibited higher levels of total macrophages (F4/80^+^), particularly M2-type (F4/80^+^ARG1^+^) but not M1-type (F4/80^+^iNOS^+^) macrophages (**Fig. 2I; Fig. S3**), along with increased *Ccl2* and *Ccl7* expression (**Fig. 2J**) relative to LKB1^KO^-Veh/LCD, LKB1^KO^-NB/LCD, and LKB1^KO^-Veh/LCD/HFCS groups. These results suggest that M2 macrophages are predominant players in exerting NB-induced LC progression in a fructose-rich environment.

We next examined the effects of NB and fructose supplementation on LC cells *in vitro*. Real-time PCR revealed significantly elevated *CCL2* and *CCL7* transcription in A549 cells treated with NB and fructose (Fru) compared to cells treated with vehicle, fructose alone, or NB alone (**Fig. 2K**). Correspondingly, THP-1 monocyte migration was markedly increased when co-cultured with A549 cells exposed to NB and fructose, relative to all other conditions (**Fig. 2L**). Similar results were observed with bone marrow-derived macrophages (BMDMs): co-culture with LLC cells treated with NB and fructose significantly enhanced BMDM migration compared to controls (**Fig. 2M**). These findings suggest that high-fructose intake promotes NB-induced tumor infiltration of M2-type macrophages.

To evaluate how both NB exposure and fructose supplementation influence the pro-tumoral activity of macrophages, we assessed the effects of conditioned media (CM) from THP-1 cells (^CM^THP) exposed to Veh or NB, with or without fructose (Fru, 10 g/L) supplementation: ^CM^THP-Veh, ^CM^THP-NB,^ CM^THP-Veh/Fru, or ^CM^THP-NB/Fru. A549 cells cultured with ^CM^THP-NB/Fru exhibited significantly greater colony-forming capacity compared to those treated with ^CM^THP-Veh, ^CM^THP-NB, or^ CM^THP-Veh/Fru (**Fig. 2N**). We next examined the effects of CM from bone marrow-derived macrophages (^CM^BMDM) exposed to Veh or NB, in the absence or presence of fructose (^CM^BMDM-Veh,^ CM^BMDM-NB,^ CM^BMDM-Veh/Fru, and ^CM^BMDM-NB/Fru) on LLC cells. LLC cells cultured with ^CM^BMDM-NB/Fru exhibited significantly enhanced colony-forming ability compared to all other groups (**Fig. 2O**). These findings suggest that NB exposure combined with fructose supplementation enhances the ability of lung cancer cells to recruit and reprogram monocytes into pro-tumoral macrophages within the TME, thereby accelerating LC progression.

### Fructose supply potentiates NB-mediated monocyte differentiation and polarization into pro-tumoral M2-like macrophages

We investigated how NB exposure programs maturing macrophages toward pro-tumoral activity under high-fructose conditions. Monocytes typically differentiate into macrophages or dendritic cells (DCs) upon tissue entry 9. NB exposure significantly increased mRNA expression of macrophage markers (*CD14* and* CD68*), but not the DC marker *CD1A* 35 in THP-1 cells (**Fig. S4A**). NB-treated THP-1 cells also upregulated M2-associated, pro-metastatic genes (*ARG1*, *CCL22*, *IL10*, *TGFB1*, and *VEGFA*) rather than M1-specific genes (*IL1B* and *NOS2*) (**Fig. S4A**). Consistently, the protein expression of ARG1, CCL22, IL-10, TGF-β, and VEGFA was also increased following NB treatment (**Fig. S4B**). These results suggest that NB promotes monocyte-to-macrophage differentiation and polarization toward a pro-metastatic M2 state.

**Figure 3:**
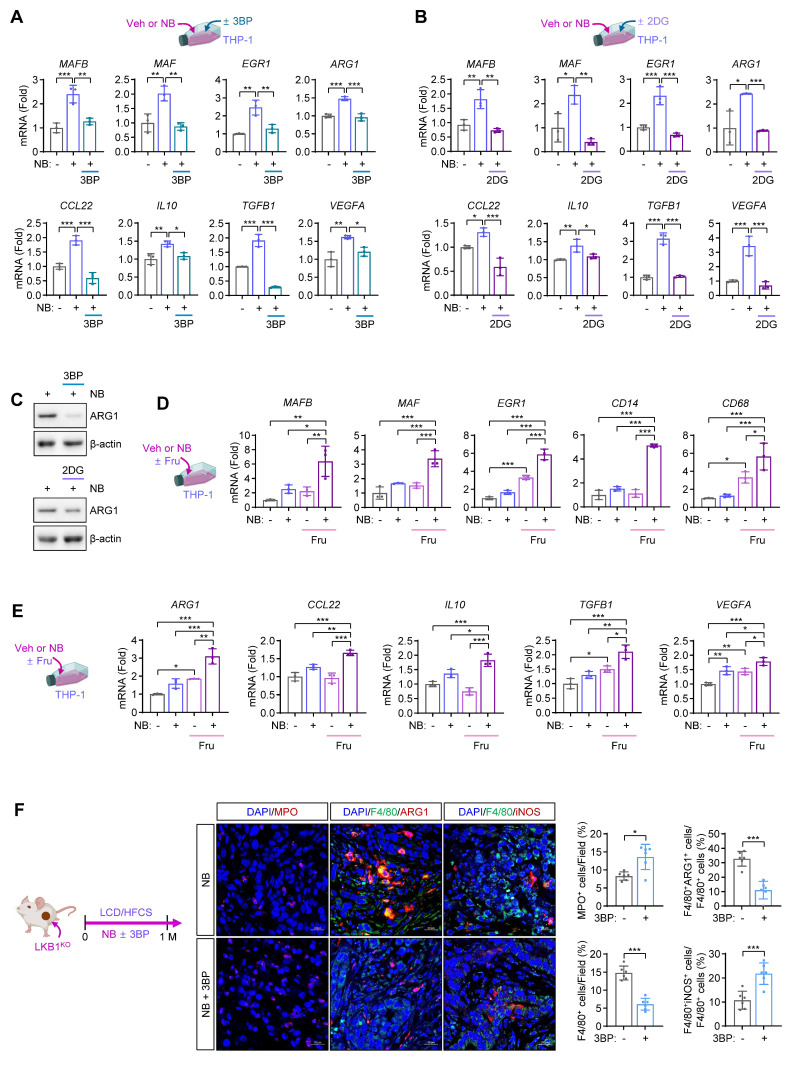
** Fructose supplementation enhances NB-induced monocyte recruitment and M2-like macrophage differentiation and polarization.** (**A-C**) THP-1 cells were treated with vehicle (Veh) or NB for three days under standard culture conditions, either alone or in combination with 3-bromopyruvate (3BP, 25 μM) or 2-deoxy-D-glucose (2DG, 5 mM). (**A, B**) Real-time PCR analysis of the indicated genes in THP-1 cells treated with NB alone or in combination with 3BP (**A**) or 2DG (**B**) (mean ± SD, *n* = 3). (**C**) Representative western blot analysis of arginase 1 (ARG1) expression in THP-1 cells treated as indicated. (**D, E**) Real-time PCR analysis of the indicated genes in THP-1 cells treated with Veh or NB for three days under low-glucose conditions (0.8 g/L glucose) with or without fructose (Fru, 10 g/L) supplementation (mean ± SD,* n* = 3). (**F**) Representative immunofluorescence images and quantitative analysis of MPO^+^ neutrophils, M1 macrophages (F4/80^+^iNOS^+^), and M2 macrophages (F4/80^+^ARG1^+^) infiltrating tumors from mice in the indicated groups (mean ± SD, *n* = 6). Scale bars, 20 μm. *p < 0.05, **p < 0.01, and ***p < 0.001, as determined by one-way ANOVA with Dunnett's multiple-comparison test (**A, B, D, E**) and a two-tailed Student's t-test (**F**).

External signals regulate monocyte fate by modulating TFs such as MAFB, MAF, and EGR1, which drive monocyte-to-macrophage differentiation 9. In this study, NB exposure induced the expression of *MAFB*, *MAF*, *EGR1*, and M2-associated genes in THP-1 cells (**Fig. S4C**). In contrast, treatment with glucose metabolism inhibitors (3BP and 2DG) suppressed NB-induced expression of these genes (**Figs. 3A, B**), without affecting *IL1B* or *NOS2* (**Fig. S4D**). Western blot analysis confirmed that 2DG and 3BP reduced ARG1 protein levels in NB-treated THP-1 cells (**Fig. 3C**).

To evaluate the impact of high-fructose supplementation on NB-mediated gene expression linked to macrophage differentiation and pro-tumoral polarization, we analyzed THP-1 cells that were treated with NB under low-glucose conditions (0.8 g/L glucose) in the presence or absence of fructose supplementation (10 g/L). We found that NB-induced expression of *MAFB*, *MAF*, *EGR1*, and M2-associated genes was further enhanced by fructose supplementation (**Figs. 3D, E**). Consistently, 3BP treatment during NB exposure in LKB1^KO^ tumor-bearing mice under LCD/HFCS condition reduced macrophage infiltration, particularly M2-type (F4/80^+^ARG1^+^), but not M1-type (F4/80^+^iNOS^+^), in tumors (**Fig. 3F**). Together, these results suggest that NB exposure under high-fructose conditions promotes monocyte differentiation and M2 polarization via TF expression and glycolytic metabolism.

### NB exposure and high-fructose supplementation cooperate to drive epigenetic reprogramming of monocyte differentiation and macrophage polarization

**Figure 4:**
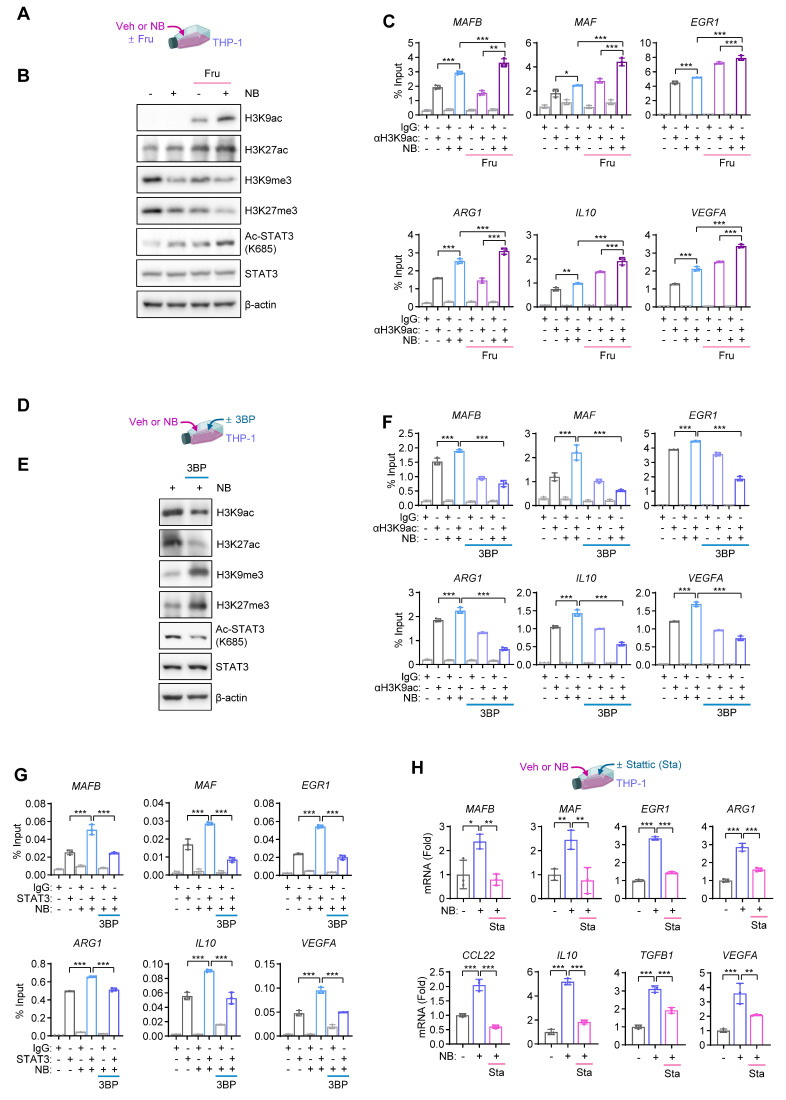
** NB promotes epigenetic upregulation of differentiation-associated transcription factors.** (**A-C**) THP-1 cells were treated with vehicle (Veh) or NB for three days under low-glucose conditions (0.8 g/L glucose) with or without fructose supplementation (Fru, 10 g/L). (**A**) Schematic diagram of the experimental design. (**B**) Representative western blot analysis images of histone H3K9 acetylation (H3K9ac), H3K27 acetylation (H3K27ac), H3K9 trimethylation (H3K9me3), H3K27 trimethylation (H3K27me3), acetylated STAT3 (Ac-STAT3), and total STAT3. (**C**) Chromatin immunoprecipitation (ChIP) assay of H3K9ac enrichment at the promoter of *MAFB*, *MAF*, *EGR1*, *ARG1*, *IL10*, or *VEGFA* in Veh- or NB-treated THP-1 cells with or without fructose supplementation (mean ± SD,* n* = 3). (**D-H**) THP-1 cells were treated with Veh or NB for three days under standard culture conditions, either alone or in combination with 3-bromopyruvate (3BP, 25 μM) or Stattic (Sta, 2 μM). (**D**) Schematic diagram of the experimental design. (**E**) Representative western blot analysis images of the indicated proteins. (**F**, **G**) ChIP analysis of H3K9ac enrichment (**F**) and STAT3 binding (**G**) to the promoters of *MAFB*, *MAF*, *EGR1*, *ARG1*, *IL10*, or *VEGFA* in Veh- or NB-treated THP-1 cells in the absence or presence of 3BP (mean ± SD,* n* = 3). (**H**) Schematic diagram of the experimental design and real-time PCR analysis of the indicated genes in THP-1 cells treated as indicated (mean ± SD,* n* = 3). *p < 0.05, **p < 0.01, and ***p < 0.001, as determined by one-way ANOVA with Dunnett's multiple-comparison test (**C**,** F**,** G, H**).

We investigated how high-fructose supplementation facilitates monocyte-to-macrophage differentiation and polarization toward an M2-type phenotype. Fructolysis shares key enzymes and intermediates with glycolysis 36, and both pathways generate acetyl-coenzyme A (acetyl-CoA), a critical substrate for histone acetylation and transcriptional activation 37. THP-1 cells were exposed to NB in the presence or absence of fructose supplementation (**Fig. 4A**). NB exposure under fructose-supplemented conditions significantly increased histone H3 acetylation at lysine 9 (H3K9ac) and lysine 27 (H3K27ac) (**Fig. 4B**). In parallel, repressive histone methylation marks (H3K9me3 and H3K27me3) were reduced, indicating a shift toward a more transcriptionally permissive chromatin state. Consistently, chromatin immunoprecipitation analysis revealed increased H3K9ac enrichment at the promoters of genes encoding differentiation-associated transcription factors (MAFB, MAF, and EGR1) and M2-associated pro-metastatic mediators (*ARG1*, *IL10*, and *VEGFA*) following NB exposure, with further enhancement upon fructose supplementation (**Fig. 4C**). Given that acetyl-CoA can also promote acetylation-dependent activation of STAT3, a transcription factor regulating both differentiation-associated TFs and M2 macrophage-related genes 38, we hypothesized that NB-enhanced fructose metabolism facilitates STAT3 activation via acetylation. Supporting this, NB exposure, particularly under fructose supplementation, increased acetylated STAT3 (Ac-STAT3) levels (**Fig. 4B**).

To determine whether this epigenetic reprogramming depends on glycolytic metabolism, we treated NB-exposed THP-1 cells with 3BP under standard culture condition (**Fig. 4D**). 3BP reduced H3K9ac and H3K27ac levels, while restoring repressive marks (H3K9me3 and H3K27me3) (**Fig. 4E**). Moreover, 3BP significantly suppressed NB-induced H3K9ac enrichment at the promoters of *MAFB*, *MAF*, *EGR1*, *ARG1*, *IL10*, and *VEGFA* (**Fig. 4F**). In addition, treatment with 3BP under the standard culture condition suppressed Ac-STAT3 expression (**Fig. 4E**) and reduced STAT3 binding to these promoters (**Fig. 4G**). Pharmacological inhibition of STAT3 with Stattic (Sta) similarly attenuated NB-induced transcription of these genes (**Fig. 4H**). Collectively, these findings indicate that NB exposure under high-fructose conditions enhances acetyl-CoA-driven histone and STAT3 acetylation, thereby activating transcriptional programs that promote monocyte differentiation and M2 macrophage polarization, contributing to a pro-metastatic phenotype.

### NB-mediated activation of β-AR, nAChR, and AhR signaling enhances fructose transporter expression in macrophages

**Figure 5:**
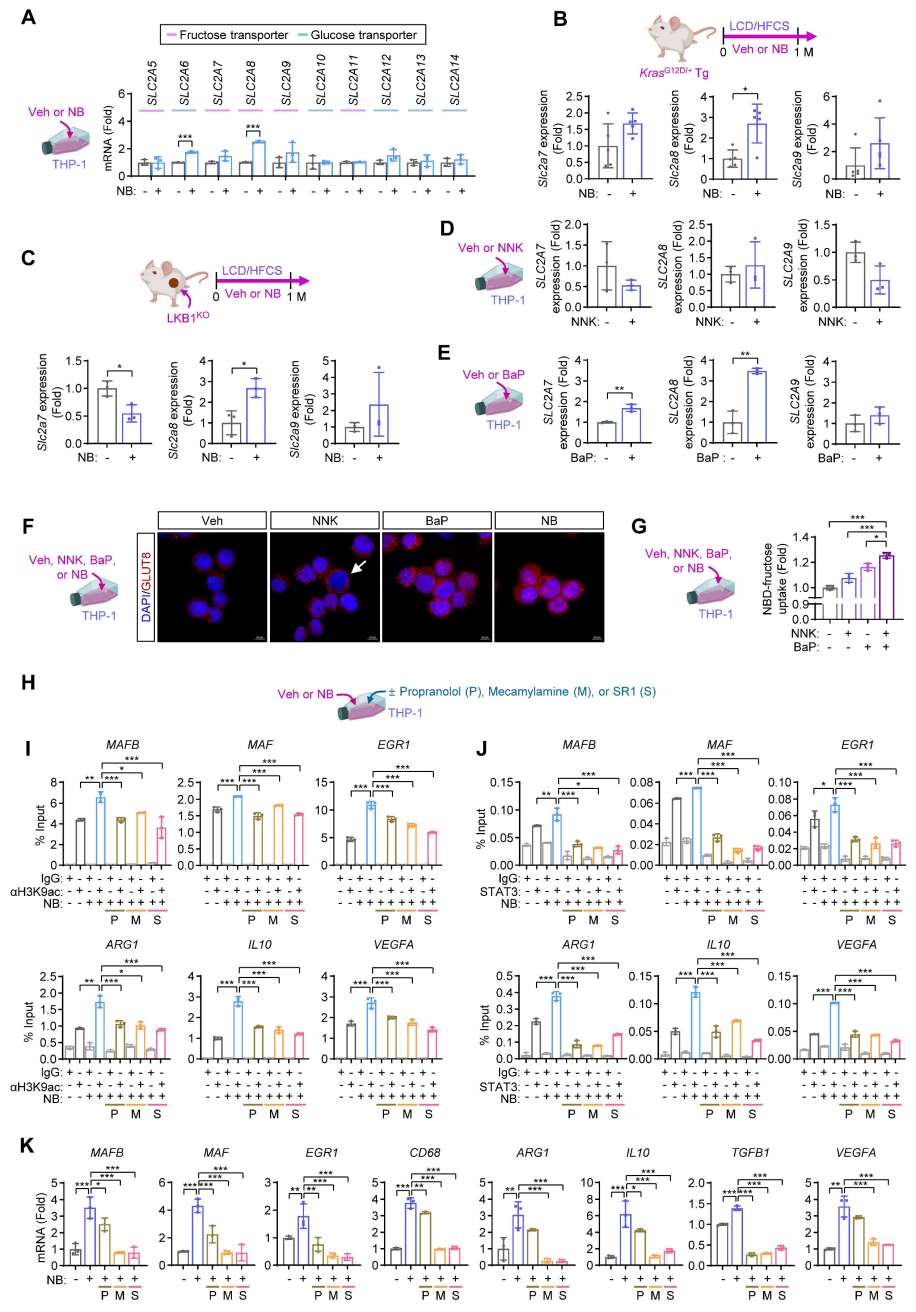
** NB enhances fructose uptake in macrophages through induction of GLUT8.** (**A**) Real-time PCR analysis of the indicated GLUTs in THP-1 cells treated with vehicle (Veh) or NB for three days under standard culture conditions (mean ± SD,* n* = 3). Except for SLC2A5/GLUT5, the GLUTs denoted as the fructose transporter have been known to transport both glucose and fructose. (**B, C**) Real-time PCR analysis of the indicated GLUTs in *Kras*^G12D/+^ Tg mouse lung tumors (**B**, mean ± SD,* n* = 5) and in LKB1^KO^ allograft tumors (**C**, mean ± SD,* n* = 3). (**D, E**) Real-time PCR analysis of the indicated GLUTs in THP-1 cells treated with NNK (1 μM, **D**) or benzo[a]pyrene (BaP, 1 μM, **E**) for three days (mean ± SD,* n* = 3). (**F**) Immunofluorescence analysis of GLUT8 subcellular localization in THP-1 cells treated with vehicle (Veh), NNK, BaP, or a combination of NNK and BaP (NB) for three days. The white arrow highlights membrane localization of GLUT8. Scale bars, 10 μm. (**G**) Quantification of NBD-fructose (20 μM) uptake in THP-1 cells treated as indicated for three days (mean ± SD,* n* = 3). (**H-K**) THP-1 cells were treated with Veh or NB for three days under standard culture conditions in the presence or absence of the indicated inhibitors (P: 10 μM propranolol; M: 10 μM mecamylamine; and S: 1 μM SR1). (**H**) Schematic diagram of the experimental design. (**I**) ChIP assay of H3K9ac enrichment at the promoters of *MAFB*, *MAF*, *EGR1*, *ARG1*, *IL10*, and *VEGFA* (mean ± SD,* n* = 3). (**J**) ChIP analysis of STAT3 binding to the same promoters (mean ± SD,* n* = 3). (**K**) Real-time PCR analysis of the differentiation-associated transcription factors, macrophage markers, and M2 macrophage markers in THP-1 cells as indicated (mean ± SD,* n* = 3). *p < 0.05, **p < 0.01, and ***p < 0.001, as determined by a two-tailed Student's t-test (**A-E**) and one-way ANOVA with Dunnett's multiple-comparison test (**G, I-K**).

Building on our previous findings that NB exposure enhances glucose uptake in TAMs via transcriptional upregulation and membrane translocation of GLUT1 and GLUT3 2, we next investigated how NB regulates fructose utilization in macrophages. We first examined the expression of glucose/fructose transporters (*SLC2A5-SLC2A14*, encoding GLUT5-GLUT14) in NB-treated THP-1 cells. NB exposure substantially increased the expression of *SLC2A7*, *SLC2A8*, and *SLC2A9*, all of which mediate glucose and/or fructose transport (**Fig. 5A**). Consistently, transcriptional upregulation of *Slc2a8* and *Slc2a9* was observed in tumors from Kras^G12D^-NB/LCD/HFCS (**Fig. 5B**) and LKB1^KO^-NB/LCD/HFCS mice (**Fig. 5C**), compared to their respective controls. To delineate the individual contributions of tobacco carcinogens, we found that BaP, but not NNK, robustly induced *SLC2A7* and *SLC2A8* expression in THP-1 cells (**Figs. 5D, E**). Immunofluorescence analysis further revealed that NNK treatment facilitated the membrane localization of GLUT8, whereas BaP induced overall GLUT8 expression (**Fig. 5F**). Notably, combined treatment with NNK and BaP (NB) enhanced both transporter expression and membrane localization, resulting in a significant additive increase in fructose uptake (**Figs. 5F, G**).

We investigated how NB exposure influences macrophage differentiation under excess fructose. NNK signals through β-AR and nAChR, while BaP activates AhR 39. Pharmacological suppression of β-AR (using propranolol [P]), nAChR (using mecamylamine [M]), or AhR (using SR1 [S]) (**Fig. 5H**) reduced NB-induced H3K9ac enrichment (**Fig. 5I**) and STAT3 binding (**Fig. 5J**) at promoters of differentiation-related TFs and M2-associated genes in THP-1 cells. This inhibition also suppressed NB-induced transcription of these genes (**Fig. 5K**). These findings suggest that BaP/AhR-, NNK/β-AR-, and NNK/nAChR-mediated signaling cooperatively enhance fructose uptake, particularly via GLUT8, leading to increased acetyl-CoA production, histone and STAT3 acetylation, and activation of transcriptional programs that drive M2 macrophage polarization.

### Expression of fructose transporters and pro-metastatic M2 macrophage markers correlates with NSCLC severity and metastasis

**Figure 6:**
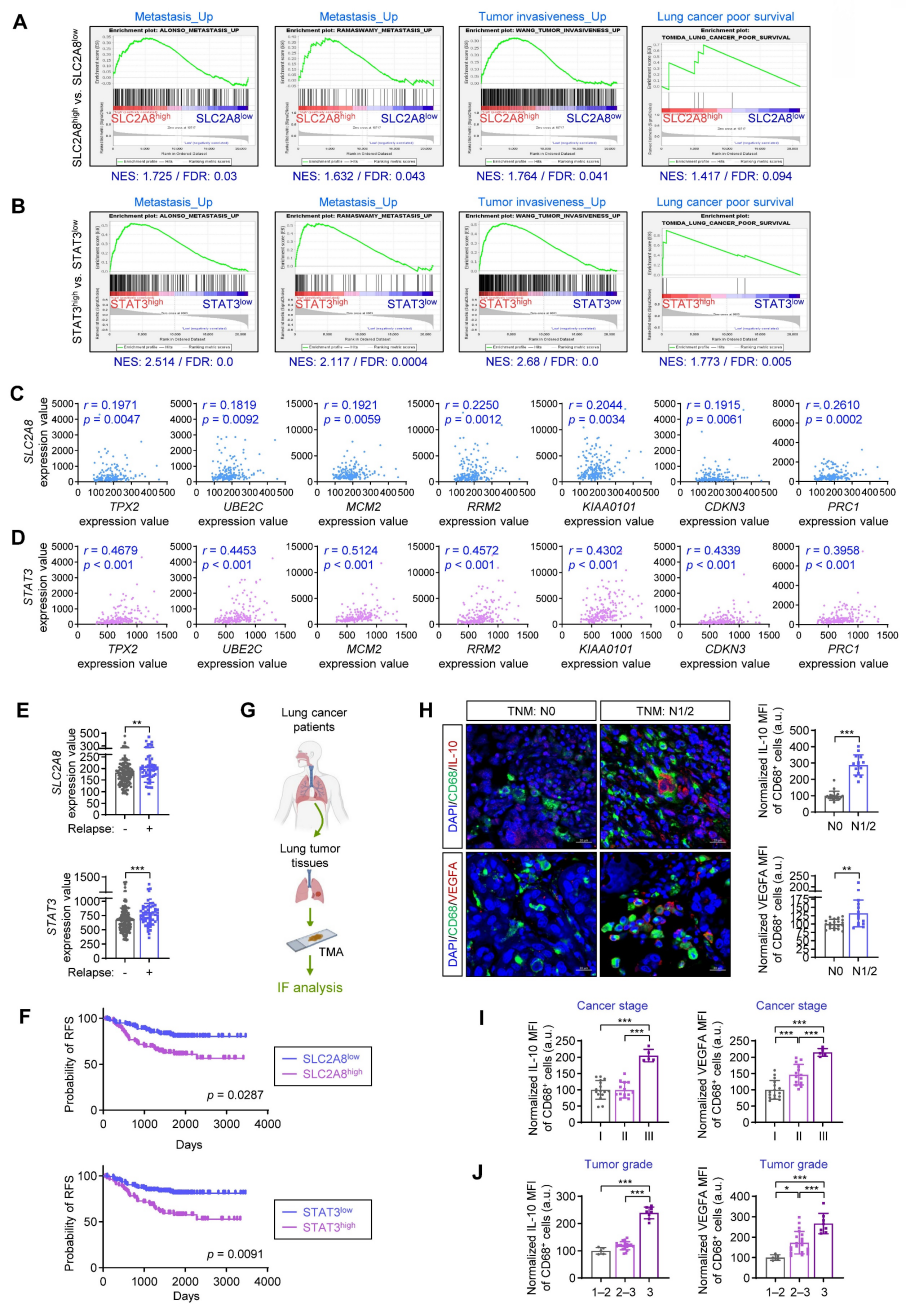
** Elevated GLUT8 or STAT3 expression and increased IL-10^+^ or VEGFA^+^ macrophages are associated with poor clinical outcomes in NSCLC.** (**A**,** B**) Gene set enrichment analysis (GSEA) of gene sets associated with metastasis, tumor invasiveness, and poor survival in lung cancer in GLUT8^high^ (**A**) or STAT3^high^ (**B**) tumors compared with their respective controls using a GSE31210 dataset. NES: normalized enrichment score. FDR: false discovery rate. (**C**,** D**) Spearman's rank correlation coefficient analysis between GLUT8 (SLC2A8, **C**) or STAT3 (**D**) expression and prognosis-associated genes in NSCLC (GSE31210, *n* = 204). (**E**) Expression levels of GLUT8 (SLC2A8) and STAT3 in NSCLC patients with or without relapse (GSE31210; no relapse: *n* = 150; relapse: *n* = 54). (**F**) Relapse-free survival (RFS) analysis according to GLUT8 or STAT3 expression in NSCLC patients (a GSE31210 dataset), assessed using the PrognoScan online platform. (**G**) Schematic diagram of the experimental design using human NSCLC tissue microarray (TMA). (**H**) Representative immunofluorescence images showing IL-10^+^ or VEGFA^+^ tumor-infiltrating CD68^+^ macrophages in NSCLC tissues without (N0, *n* = 21) or with lymph node metastasis (N1/2, *n* = 14) according to TNM staging. Scale bars, 20 μm. (**I**,** J**) Association between marker expression and cancer stage (**I**; stage I, *n* = 16; stage II, *n* = 14; stage III, *n* = 5) or tumor grade (**J;** grade 1-2, *n* = 5; grade 2-3, *n* = 20; grade 3, *n* = 8). *p < 0.05, **p < 0.01, and ***p < 0.001, as determined by Mann-Whitney test (**E, H**), a two-tailed Student's t-test (**H**), and one-way ANOVA with Tukey's multiple-comparison test (**I**,** J**).

To evaluate the clinical relevance of GLUT8 and STAT3 in NSCLC, we performed gene set enrichment analysis (GSEA) using the GSE31210 dataset. Gene sets associated with metastasis, tumor invasiveness, and lung cancer poor survival were significantly enriched in the SLC2A8^high^ (**Fig. 6A**) and STAT3^high^ (**Fig. 6B**) groups compared with their respective low-expression counterparts (FDR < 0.25). Correlation analysis using a panel of poor-prognosis genes in lung adenocarcinoma or squamous cell carcinoma (*TPX2*, *UBE2C*, *MCM2*, *RRM2*, *KIAA0101*, *CDKN3*, *PRC1*, *FGFR2*, and *GPC3*) 25 revealed significant positive correlations between *SLC2A8* or *STAT3* and seven of these genes (*TPX2*, *UBE2C*, *MCM2*, *RRM2*, *KIAA0101*, *CDKN3, and PRC1*) (**Figs. 6C, D**). Consistently, *SLC2A8* and *STAT3* expression levels were significantly higher in NSCLC patients who experienced relapse (**Fig. 6E**), and elevated expression of either gene was associated with shorter relapse-free survival (**Fig. 6F**).

We further evaluated IL-10 and VEGFA expression in CD68^+^ macrophages using a tissue microarray (TMA) comprising 70 NSCLC tumor cores obtained from 35 patients, annotated with clinicopathological parameters including TNM stage, cancer stage, and tumor grade (**Fig. 6G**). IL-10 and VEGFA expression in CD68^+^ macrophages were significantly higher in tumors from patients with lymph node metastasis (N1/2) compared to those without nodal involvement (N0) (**Fig. 6H**). Consistently, the expression of these markers was higher in advanced-stage cancer (stage III) (**Fig. 6I**) and in high-grade tumors (grades 2-3 or 3) (**Fig. 6J**) compared to early-stage cancer (stage I) and low-grade tumors (grade 1-2). These findings indicate the association of elevated SLC2A8/STAT3 signaling with a poor prognosis in NSCLC. Additionally, these results suggest that IL-10^+^ and VEGFA^+^ M2 macrophages may serve as potential biomarkers of disease severity and metastatic potential.

## Discussion

**Figure 7:**
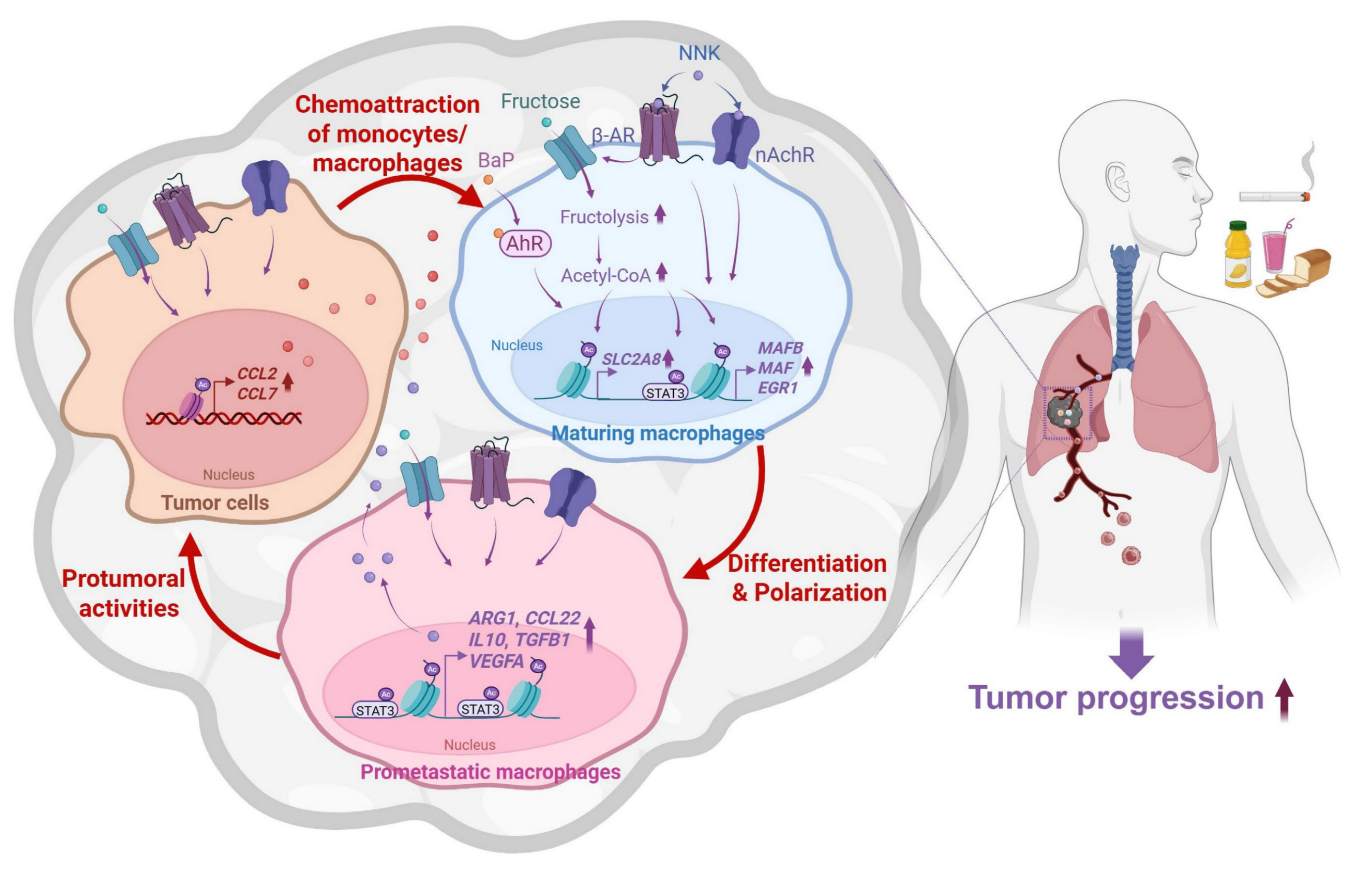
** Proposed model of NB-induced lung cancer progression mediated by macrophage reprogramming in a fructose-rich microenvironment.** NB promotes macrophage recruitment by inducing chemokine expression in tumor cells. Under high-fructose conditions, NB enhances fructose utilization in recruited macrophages, leading to acetyl-CoA-dependent epigenetic reprogramming. This process induces differentiation-associated transcription factors and M2 macrophage-associated genes, thereby driving polarization toward an M2-like phenotype. These educated macrophages subsequently promote tumor progression and metastasis of lung cancer cells.

Dietary factors and TS are known to influence cancer development; however, their cooperative impact on LC progression remains incompletely understood. In this study, we demonstrate that excessive fructose intake markedly accelerates NB-induced LC progression in mouse models. Under fructose-rich conditions, NB exposure upregulated chemokines that promote monocyte recruitment into the tumor microenvironment and facilitated their differentiation into pro-tumoral macrophages. Mechanistically, NB-activated AhR, nAChR, and β-AR signaling enhanced fructose uptake through transcriptional and post-transcriptional upregulation of fructose transporters. This metabolic reprogramming increased acetyl-CoA production, leading to enhanced acetylation of histones and STAT3. These epigenetic alterations induced differentiation-associated transcription factors (MAFB, MAF, and EGR1) and M2 macrophage-associated genes, thereby driving polarization toward a pro-metastatic phenotype (**Fig. 7**). Collectively, our findings reveal a cooperative interaction between dietary fructose and tobacco carcinogens in metabolically and epigenetically reprogramming macrophages, ultimately promoting LC progression.

High-carbohydrate diets (HCDs) have been associated with cancer progression, whereas ketogenic diets lower circulating glucose levels and have been shown to suppress tumor growth 40. Notably, in patients with a history of LC or TS, post-treatment ketogenic diets were associated with reduced brain metastasis without relapse 41. Consistent with these observations, we previously demonstrated that NB exposure accelerates LC progression in a glucose-rich environment 2. Although glucose metabolism is a well-established driver of tumor growth, fructose can similarly support cancer progression by fueling energy production, nucleotide biosynthesis, and lipogenesis 42. In the present study, we show that excessive fructose intake markedly enhances NB-induced LC growth and metastasis, including in KRAS- and LKB1-mutant models, consistent with prior experimental and clinical findings 42. Mechanistically, NB exposure under high-fructose conditions upregulated chemokines that promote macrophage recruitment and increased infiltration of M2-like TAMs. These TAMs likely facilitate LC progression through the secretion of soluble mediators that enhance tumor-microenvironment crosstalk and activate oncogenic signaling pathways in cancer cells 43. Supporting this macrophage-dependent mechanism, our previous work demonstrated that clodronate liposome-mediated macrophage depletion significantly suppressed NB-driven LC progression in HCD-fed mice 44.

A key question is how NB exposure and an HCD influence monocyte fate within the tumor microenvironment (TME). Acetyl-CoA production has been shown to drive histone modifications that regulate monocyte differentiation 9. Consistent with this concept, we found that NB exposure under fructose supplementation increased histone and STAT3 acetylation, leading to transcriptional activation of key differentiation-associated transcription factors and promoting monocyte-to-macrophage differentiation. We next investigated how NB enhances fructose utilization in TAMs. Our data indicate that BaP-mediated signaling induces GLUT8 expression during macrophage maturation, whereas NNK-mediated signaling facilitates GLUT8 membrane localization. This coordinated action of BaP and NNK enhances fructose uptake, resulting in increased acetyl-CoA availability and subsequent acetylation of histones and STAT3—paralleling their previously described effects on GLUT1 and GLUT3 regulation 2. This epigenetic reprogramming activates transcriptional programs that drive macrophage differentiation and pro-tumoral function. Although additional pathways may contribute, our findings support a model in which NB-induced metabolic and epigenetic remodeling promotes M2 polarization, thereby facilitating LC progression. Notably, GLUT8 emerges as a central mediator in this axis and represents a potential therapeutic target in LC.

Previous studies have reported an association between elevated blood glucose levels and increased cancer risk 45. In contrast, the relationship between circulating fructose levels and cancer remains poorly defined. In healthy fasting humans, circulating plasma fructose is extremely low, typically around 0.008 to 0.05 mM, roughly 100-fold lower than fasting blood glucose concentrations (~5.5 mM) 46-48. Even after acute fructose ingestion, peripheral fructose levels generally remain within the low micromolar range, as most absorbed fructose is rapidly extracted by the liver, with concentrations returning to baseline within approximately two hours 49, 50. Data on circulating fructose levels in cancer patients are limited and inconsistent. Although one report described elevated fasting serum fructose levels in patients with pancreatic cancer, this observation has not been systematically replicated across other tumor types, including lung cancer 51, 52. Consequently, no well-defined “pathological” systemic fructose threshold has been established for either healthy individuals or cancer patients. Rather than systemic elevation, accumulating evidence—including our findings—supports a model in which cancer cells and tumor-associated immune cells selectively exploit fructose locally, even at low circulating concentrations. Tumor cells can upregulate the fructose transporter GLUT5 (SLC2A5) and generate fructose de novo from glucose via the polyol pathway, thereby sustaining intracellular fructose metabolism without requiring marked systemic increases 53. In our study, we further identify increased expression of GLUT8 (SLC2A8) in TAMs and demonstrate that this facilitates fructose utilization and pro-tumoral macrophage polarization. Thus, although systemic fructose remains low, a high-fructose diet combined with transporter upregulation in tumor cells (GLUT5) and macrophages (GLUT8) may establish a localized fructose-enriched metabolic niche that promotes tumor progression.

The mechanistic insights into the NB-induced LC progression have important translational implications. Accelerated tumor progression, poor prognosis, and reduced therapeutic efficacy are frequently observed in cancer patients with hyperglycemia, commonly associated with diabetes, HCDs, and/or obesity 54, 55. These observations underscore the clinical relevance of metabolic reprogramming in cancer progression. From a therapeutic perspective, strategies aimed at glucose restriction, inhibition of glycolysis, or depletion of TAMs may help limit the pro-metastatic potential of NSCLC cells and mitigate TS-associated progression. Our findings further suggest that GLUT8 expression in TAMs may serve both as a prognostic biomarker and as a therapeutic target in LC patients with persistent tobacco exposure. Nevertheless, this study has limitations. Although several key findings were supported by analyses of publicly available datasets and human tissue microarrays, the mechanistic conclusions are based primarily on NB exposure models, cell lines, and murine systems. Additional clinical studies and prospective validation will be required to establish the therapeutic feasibility and prognostic value of targeting GLUT8 in TAMs in patients with NSCLC.

Despite these limitations, our findings uncover a mechanistic link between excessive carbohydrate intake, including fructose, and TS-induced LC progression. Through complementary *in vivo* and *in vitro* approaches, we delineate pathways by which NB exposure enhances tumor infiltration and epigenetically reprograms maturing macrophages toward pro-tumoral phenotypes. If translatable to humans, a high-carbohydrate or high-sugar diet combined with persistent tobacco exposure may accelerate disease progression. Future clinical investigations should evaluate systemic fructose levels in LC patients with or without tobacco exposure and determine how dietary sugar intake influences clinical outcomes.

## Supplementary Material

Supplementary figures and tables.
