# Supplementary material for: Monocyte Preprogramming by Tobacco Carcinogens and Fructose Intake Accelerates Lung Cancer Progression via Metabolic and Epigenetic Pathways
Source: Int J Biol Sci. 2026 Mar 25;22(7):3788–806. doi: 10.7150/ijbs.125622 (PMC13086078; doi:10.7150/ijbs.125622)
Supplement: Supplementary file 1 — Supplementary figures and tables. [file ijbsv22p3788s1.pdf]

Supplementary Information

Monocyte Preprogramming by Tobacco Carcinogens and Fructose Intake Accelerates Lung Cancer Progression via Metabolic and Epigenetic Pathways

Table S1. Primer sequences used for real-time PCR.

Species	Gene	Forward sequence (5´-3´)	Reverse sequence (5´-3´)
Human	*CD1A*	ATACGCACCATTCGGTCATTT	GCTCACAAAGTCTGATCCTTGAT
Human	*CD14*	ACGCCAGAACCTTGTGAGC	GCATGGATCTCCACCTCTACTG
Human	*CD68*	CTTCTCTCATTCCCCTATGGACA	GAAGGACACATTGTACTCCACC
Human	*ARG1*	TGGACAGACTAGGAATTGGCA	CCAGTCCGTCAACATCAAAACT
Human	*CCL22*	CCCTACGGCGCCAACAT	CAGACGGTAACGGACGTAATCA
Human	*IL10*	GAACCAAGACCCAGACATC	CATTCTTCACCTGCTCCAC
Human	*TGFB1*	GACACCAACTATTGCTTCAG	CAGGCTCCAAATGTAGGG
Human	*VEGFA*	TTGCCTTGCTGCTCTACCTCCA	GATGGCAGTAGCTGCGCTGATA
Human	*MAFB*	TCAAGTTCGACGTGAAGAAGG	GTTCATCTGCTGGTAGTTGCT
Human	*MAF*	GAGACCGACCGCATCATCAG	GGTAGCCGGTCATCCAGTAG
Human	*EGR1*	GGTCAGTGGCCTAGTGAGC	GTGCCGCTGAGTAAATGGGA
Human	*IL1B*	TGATGGCTTATTACAGTGGCAATG	GTAGTGGTGGTGGGAGATTCG
Human	*NOS2*	GCTCTACACCTCCAATGTGACC	CTGCCGAGATTTGAGCCTCATG
Human	*SLC2A5*	ACGTTGCTGTGGTCTGTAACC	CATTAAGATCGCAGGCACGATA
Human	*SLC2A6*	CCGGACTACGACACCTTCC	GGATGTGTAGACCAGGGCATA
Human	*SLC2A7*	CAGTACGGCTACAACCTCTCT	TTGCGTGTCGCTCAAAGTAGG
Human	*SLC2A8*	CTAGTGGCCCCGGTCTACAT	CCGACGACGACCATTAGCTG
Human	*SLC2A9*	CAATAGACCCAGACACTCTGACT	TCTTCACAATTAACGTCCCCAC
Human	*SLC2A10*	CTTGCTGTATCTACGTGTCAGA	CCAGCCAGTGCATAGTTGAGG
Human	*SLC2A11*	GGAGTCAATGCAGGTGTGAG	CCAGAGCCGTAAAGATGGCTG
Human	*SLC2A12*	GAGGCTGCGGCATGTTTAC	CCAAGTTCATAACCCACCAGG
Human	*SLC2A13*	ACATTGCGGAGGTCTCACC	AGGCTCCATCAACAACACTTG
Human	*SLC2A14*	CTGCTCACGAATCTCTGGTCC	GCCTAATAGCACCGGCCATAG
Human	*ACTB*	TCATTCCAAATATGAGATGCGTTG	TAGAGAGAAGTGGGGTGGCT

Mouse	*Sox2*	GCGGAGTGGAAACTTTTGTCC	CGGGAAGCGTGTACTTATCCTT
Mouse	*Aldh1a1*	ATACTTGTCGGATTTAGGAGGCT	GGGCCTATCTTCCAAATGAACA
Mouse	*Ccl2*	TTAAAAACCTGGATCGGAACCAA	GCATTAGCTTCAGATTTACGGGT
Mouse	*Ccl7*	CAGAAGGATCACCAGTAGTCGG	ATAGCCTCCTCGACCCACTTCT
Mouse	*Slc2a7*	GATTCTCCTGCTGTCTGGCTAT	GATGGATGGAAACGTCAACC
Mouse	*Slc2a8*	TTCATGGCCTTTCTAGTGACC	GAGTCCTGCCTTTAGTCTCAG
Mouse	*Slc2a9*	TGCTTCCTCGTCTTCGCCACAATA	CTCTTGGCAAATGCCTGGCTGATT
Mouse	*Gapdh*	GAGTTGCTGTTGAAGTCGCA	GGTGGTGAAGCAGGCATCTG

Table S2. Primer sequences used for chromatin immunoprecipitation assay*

Name	Forward sequence (5´-3´)	Reverse sequence (5´-3´)
ARG1_H3K9ac	GATTCTACAATTATTTTCCTG	CATGAGGGTAAATGGTTAATC
ARG1_STAT3	GAAATGTGTCTCATGGATTAAC	CGTCCTTGTAGAAGAAGGGCC
IL10_STAT3/H3K9ac	GGAGGAGCTCTAAGCAGAA	AAGCCCCTGATGTGTAGAC
VEGFA_STAT3/H3K9ac	CTTCCCGTTCTCAGCTCCACAAAC	CTGGCCTGCAGACATCAAAGTGAG

*We conducted a chromatin immunoprecipitation assay for evaluating the binding of acetylated H3 at the lysine 9 residue (H3K9ac) or STAT3 to the *MAFB*, *MAF*, or EGR1 promoters by using primers available in the previous report (DOI: https://doi.org/10.1016/j.molcel.2020.05.004).

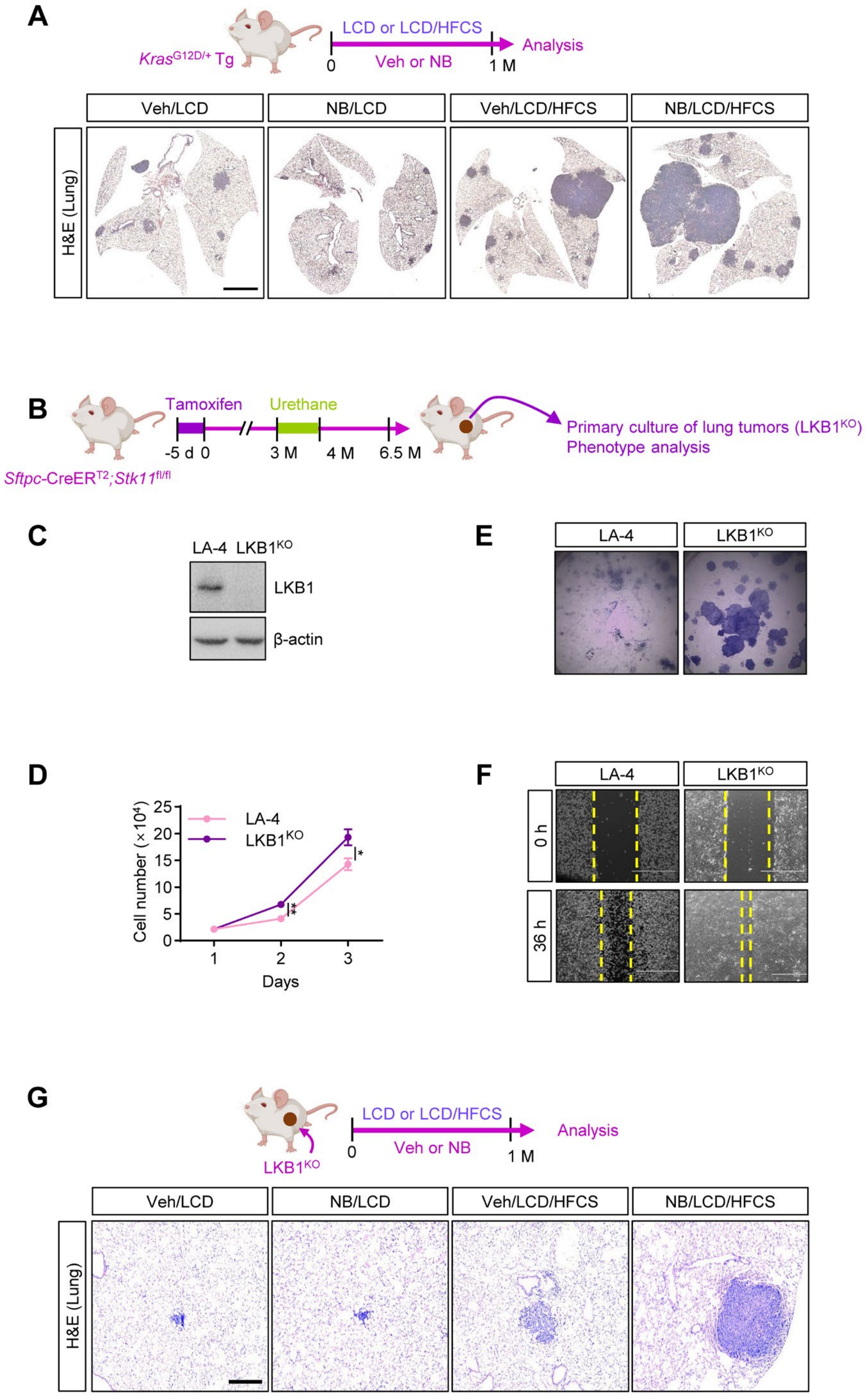

Figure S1. High-fructose corn syrup (HFCS) accelerates the 4-(methylnitrosamino)-1-(3-pyridyl)-1-butanol- and benzo[a]pyrene (NB)-induced lung tumorigenesis and characterization of LKB1^{KO} cells. (**A**) Representative images of H&E-stained lung tissues from *Kras*$^{G12D/+}$ Tg mice treated with either vehicle (Veh) or NB and fed a low-carbohydrate diet (LCD) or an LCD supplemented with high fructose corn syrup (HFCS, LCD/HFCS). Scale bar, 3 mm. (**B**) Schematic diagram illustrating the experimental protocol to establish LKB1^{KO} primary lung cancer cells. (**C**) Representative western blot analysis images for evaluating the level of LKB1 expression in LKB1^{KO} cells in comparison with LA-4 cells. (**D**) Changes in cell proliferation of LA-4 and LKB1^{KO}

cells, as determined by cell counting assay (mean ± SD, $n = 3$). $^*p < 0.05$ and $^{**}p < 0.01$, as determined by a two-tailed Student's *t*-test. (**E**) Representative images showing changes in anchorage-dependent colony formation in $LKB1^{KO}$ cells in comparison with LA-4 cells, as determined by anchorage-dependent colony formation assay. (**F**) Representative images showing changes in the migratory ability of $LKB1^{KO}$ cells compared to LA-4 cells, as determined by a scratch assay. Scale bars, 1000 μm. (**G**) Representative images of H&E-stained lung tissues of the indicated groups. Scale bar, 200 μm.

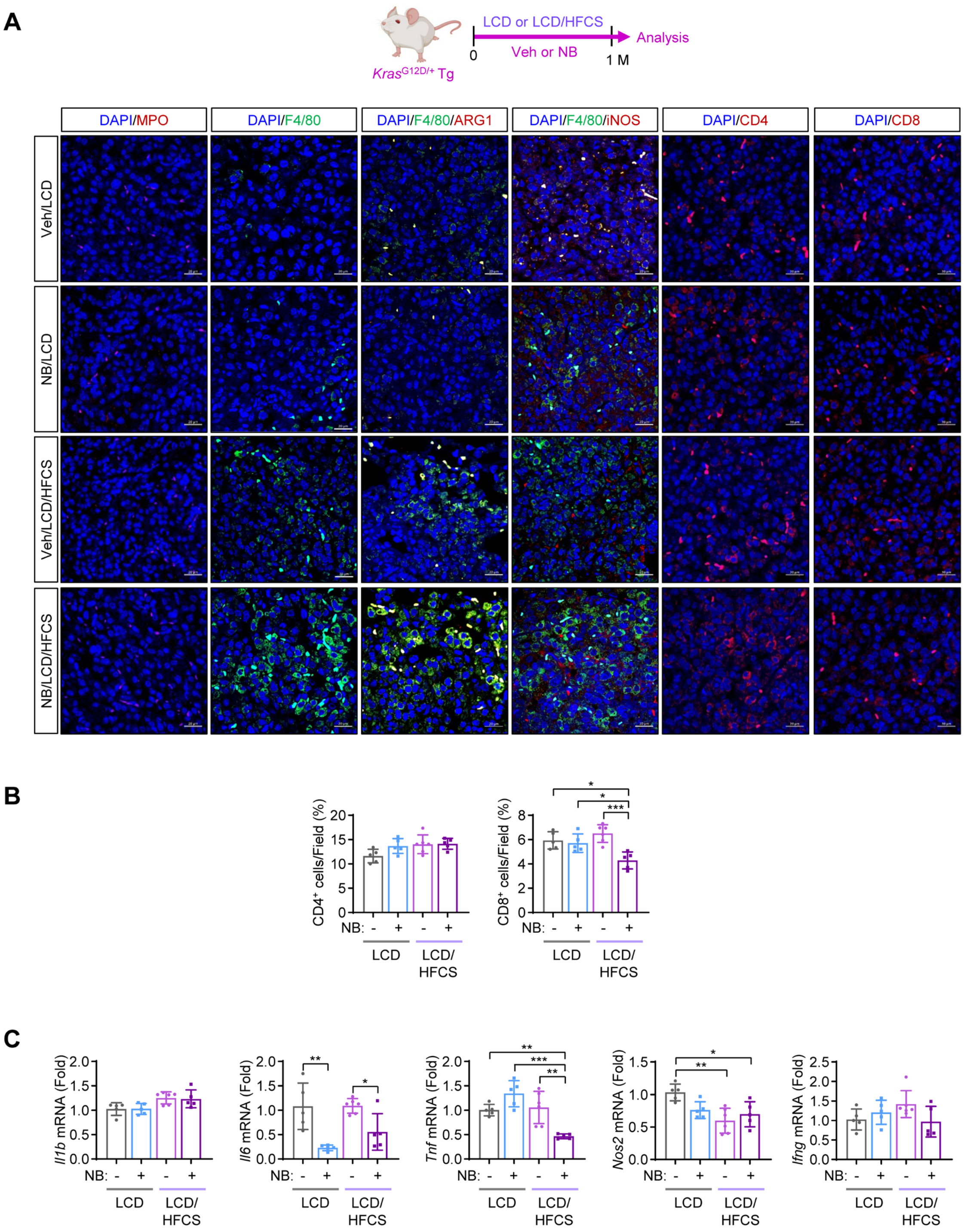

Figure S2. Fructose supplementation promotes monocyte differentiation and M2 polarization in the *Kras*$^{G12D/+}$ transgenic (Tg) mouse model. (**A**) Schematic diagram illustrating the experimental timeline and representative immunofluorescence images showing infiltration of MPO$^+$ neutrophils, F4/80$^+$ macrophages, M1 macrophages (F4/80$^+$iNOS$^+$), M2 macrophages (F4/80$^+$ARG1$^+$), CD4$^+$ T cells, and CD8$^+$ T cells in lung tumors from *Kras*$^{G12D/+}$ Tg mice treated with vehicle (Veh) or NB and fed a low-carbohydrate diet (LCD) or an LCD supplemented with high fructose corn syrup (HFCS, LCD/HFCS). Quantification of MPO$^+$, F4/80$^+$, F4/80$^+$iNOS$^+$, and F4/80$^+$ARG1$^+$ cells is presented in **Fig. 2E**. Scale bars, 20 μm. (**B**) Quantification of CD4$^+$ and CD8$^+$

T cells (mean ± SD, n = 5 or 6). (**C**) Real-time PCR analysis of the indicated genes in lung tumors derived from the specified groups of *Kras*$^{G12D/+}$ Tg mice (mean ± SD, n = 5 or 6). *$p < 0.05$, **$p < 0.01$, and ***$p < 0.001$, as determined by one-way ANOVA with Dunnett's multiple-comparison test (**B**, **C**).

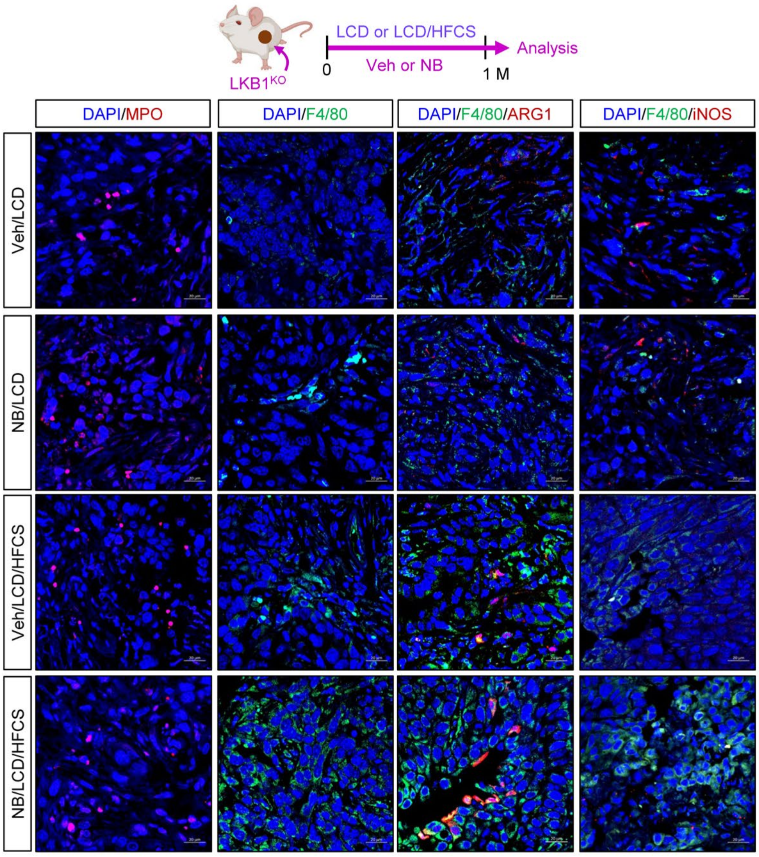

Figure S3. Fructose supplementation promotes the differentiation and polarization of monocytes into M2 macrophages in the LKB1^{KO} allograft model. Schematic diagram illustrating the experimental timeline and representative immunofluorescence images showing the infiltration of MPO$^+$ neutrophils, F4/80$^+$ macrophages, M1 macrophages (F4/80$^+$iNOS$^+$), and M2 macrophages (F4/80$^+$ARG1$^+$) in subcutaneous LKB1^{KO} tumors from mice treated with vehicle (Veh) or NB under the conditions of LCD or LCD/HFCS. Quantitative analysis results are presented in **Fig. 2I**. Scale bars, 20 μm.

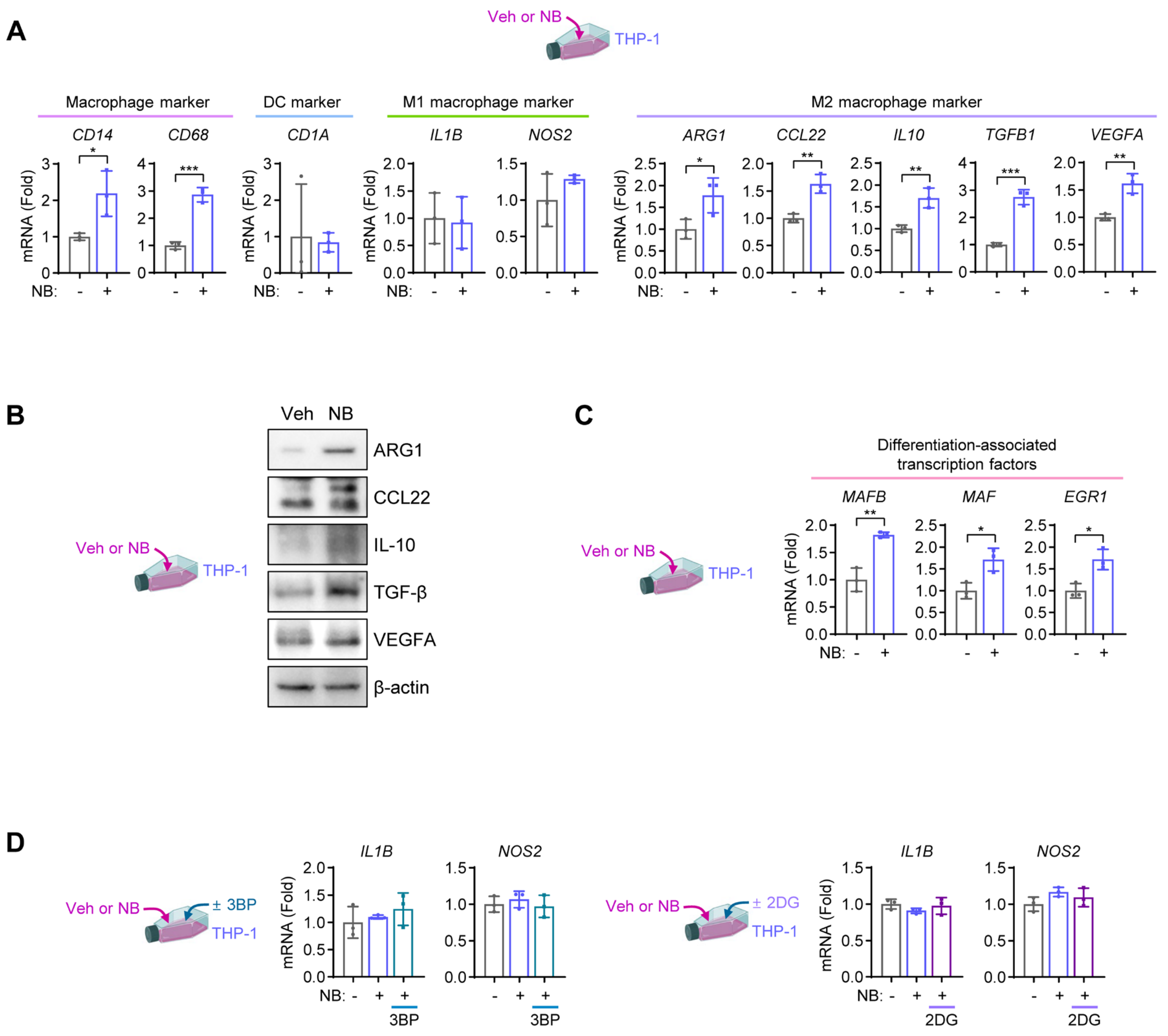

Figure S4. 4-(methylnitrosamino)-1-(3-pyridyl)-1-butanol- and benzo[a]pyrene (NB) induces the expression of M2 macrophage-associated markers. THP-1 cells were treated with NB for three days under standard culture conditions, either alone or in combination with 3-bromopyruvate (3BP, 25 μM) or 2-deoxy-D-glucose (2DG, 5 mM). (**A**) Real-time PCR analysis of mRNA expression levels of macrophage markers, dendritic cell (DC) markers, and M1- and M2-associated genes in THP-1 cells with or without NB treatment (mean ± SD, *n* = 3). (**B**) Western blot analysis of ARG1, CCL22, IL-10, TGF-β, and VEGFA expression in THP-1 cells with or without NB treatment. (**C**) Real-time PCR analysis of differentiation-associated transcription factors (*MAF*B, *MAF*, and *EGR1*) in THP-1 cells with or without NB treatment (mean ± SD, *n* = 3). (**D**) Real-time PCR analysis of the indicated genes in THP-1 cells treated with NB, either alone or in combination with 3BP or 2DG (mean ± SD, *n* = 3). *$p < 0.05$, **$p < 0.01$, and ***$p < 0.001$, as determined by a two-tailed Student's *t*-test (**A, C**). The differences among groups were not significant, as determined by one-way ANOVA with Tukey's multiple-comparison test (**D**).
